# An Ethnobotanical Study of Medicinal Plants in Kinmen

**DOI:** 10.3389/fphar.2021.681190

**Published:** 2022-02-09

**Authors:** Shyh-Shyun Huang, Chia-Hung Huang, Chien-Yu Ko, Ting-Yang Chen, Yung-Chi Cheng, Jung Chao

**Affiliations:** ^1^ School of Pharmacy, China Medical University, Taichung, Taiwan; ^2^ Department of Food Nutrition and Health Biotechnology, Asia University, Ministry of Health and Welfare, Taichung, Taiwan; ^3^ Department of Pharmacy, Kinmen Hospital, Ministry of Health and Welfare, Kinmen, Taiwan; ^4^ Department of Nursing, National Quemoy University, Kinmen, Taiwan; ^5^ Department of Pharmacology, Yale University School of Medicine, New Haven, CT, United States; ^6^ Chinese Medicine Research Center, Department of Chinese Pharmaceutical Sciences and Chinese Medicine Resources, Master Program for Food and Drug Safety, China Medical University, Taichung, Taiwan

**Keywords:** ethnobotany, historical source, field investigation, quemoy traditional medicine, taiwan traditional medicine

## Abstract

Kinmen is an outlying island that has the richest plant resources in Taiwan. The objective of this study was to record the methods that people in Kinmen use medicinal plants and to analyze the cultural characteristics of their use. Field investigations were carried out in various towns and villages in Kinmen, and 80 respondents were included in the survey. The search for respondents was conducted through local elderly people and medicinal plant groups. Semi-structured interviews were conducted with the local people to obtain their knowledge of medicinal plants and how they disseminate this information. Informed consent was obtained prior to the interviews, and the following was determined: plant use value (UV), frequency of citation (FC), and factor of informant consensus (Fic). These parameters were used to quantify the data and measure the agreement among the respondents on using plants to treat different diseases. Finally, the survey results were compared with the representative ethnobotanical literature in neighboring areas to evaluate the similarity between plant usage in Kinmen and neighboring areas as well as to determine whether there are new species or novel usages in the study area. In the Kinmen area, phytotherapy is generally used by elderly people with low educational attainments. According to the survey results, 83 medicinal plants belonging to 48 families were collected. These medicinal plants were mainly distributed in the Compositae, Lamiaceae*,* and Solanaceae families. Eighteen novel uses that have not been previously documented were found, four of which were related to newly recorded medicinal plant species in the Kinmen area. The results showed that 93.98 and 65.06% of the species collected in the present study were also recorded in literature from Taiwan and Fujian, respectively. This study showed that Kinmen’s ethnobotanical knowledge is closely related to the *Catalogue of Medicinal Plant Resources in Taiwan*, and local people indeed shared similar uses of medicinal species with people in Taiwan and Fujian (46.99%). The results from this study highlighted the importance of traditional medicine in the Kinmen area, where people have a specific understanding of using medicinal plants and communication with people in Taiwan and Fujian Province in China. It was found that Kinmen shares ethnobotanical knowledge with Taiwan and Fujian.

## 1 Introduction

Kinmen (Quemoy) is located on the west coast of the Pacific Ocean, specifically in the west-central island area of Taiwan’s main island (Chen, 2017). At present, its correct legal name is Kinmen County, and it is under the jurisdiction of Fujian Province, the Republic of China. A narrow part of the sea separates it from Xiamen City in Fujian Province, making it an independent island surrounded by the sea.

Because of its special political situation due to geographical location, population migration, war, and cultural exchange, Kinmen has been in different positions in the past, depending on the regimes and dynasties that ruled the area. According to literature records, Kinmen was influenced by ancient China early on. Although Kinmen and Taiwan are governed by the same regime at present, from a historical perspective, there is not much similarity between them (Chen, 2017). However, several battles took place in Kinmen, which is why it shares a common destiny with Taiwan (Table 1) (Sung, 2009; Sung, 2016).

**TABLE 1:** The important historical events that promoted ethnobotanical plant exchange in Kinmen.

The Eastern Jin Dynasty (A.D. 317–420)	Since the occurrence of frequent wars in the central plains during the Eastern Jin Dynasty (A.D. 317–420), people have taken refuge and moved to Kinmen because of its special geographical location
	Therefore, the migrant population has influenced the use of plants in the kinmen area for the first time in history (Li, 2009)
The Ming Zheng period (A.D. 1662–1683)	In the Ming Zheng period (A.D. 1662–1683), Zheng Chenggong took Kinmen as the base for rebelling against the Qing Dynasty and rebuilding the Ming Dynasty
	Therefore, a large number of people in Southern Fujian migrated to Kinmen along with the army (Li, 2005)
The Qing Dynasty (A.D. 1820–1850)	After the Opium War in the Daoguang Period of the Qing Dynasty (A.D. 1820–1850), five ports in China were opened for international trading and a large number of native people moved to Southeast Asia, making Kinmen the hometown of Southern Fujian culture as well as the hometown of overseas Chinese This spread the use of Kinmen plants overseas and introduced Southern Fujian culture and Nanyang folk customs to Kinmen (Li, 2009)
The period of Republic of China (A.D. 1911–1945)	After the Revolution of 1911 (A.D. 1911), China was governed by the Republic of China. At that time, Kinmen belonged to the Republic of China, while Taiwan was under the jurisdiction of Japan (Lee, 2010)
	During this period, the Japanese conducted a complete ethnobotanical survey of Taiwan, including plants used by the indigenous people
The civil war between the Kuomintang and the Communist Party (A.D. 1945–1950)	Before the end of the civil war between the Kuomintang and the Communist Party (A.D. 1950), life in Kinmen was closely connected with that in Fujian Province, but after the end of this civil war, the Republic of China withdrew to Taiwan, and Kinmen became the front line for defending the country During the Civil War between Kuomintang and the Communist Party, the influence of the war led to Kinmen establishing close and sometimes antagonistic relations with China and Taiwan, but the exchanges between soldiers from other provinces and Kinmen residents were more frequent This state brought more traditional medical knowledge into Kinmen
The period of Republic of China in Taiwan (since A.D. 1949)	The Millennium (A.D. 2000) marked the opening of Mini Three Links (Fujian Province of the People’s Republic of China and Kinmen County of Fujian Province of the Republic of China became engaged in trade, postal service, and navigation; mainland people were allowed to travel between China and Taiwan via Kinmen). It not only affected food, clothing, housing, and transportation, but also increased the cultural exchanges of Kinmen ethnic groups, thus affecting the residents’ ways of using plants (Wu, 2004)

The use of traditional phytotherapy in Kinmen has a long and rich history and is an indispensable part of their local culture because of its special geographical location and lack of medical resources. However, the use of phytotherapy in this area lacks written records and systematic arrangement. As indicated by most ethnobotanical research, we found that the traditional knowledge about medicinal plants in Kinmen is rapidly disappearing (Reyes-Garcia et al., 2013) and may gradually be lost in the future.

According to the *Flora of Kinmen*, there are currently 820 vascular plants indigenous and introduced to Kinmen, including 52 ferns, one gymnosperm, 529 dicotyledons, and 238 monocotyledons. Among them, 648 species are shared with Fujian, accounting for 92.1% of all species, and 633 species are shared with Taiwan, accounting for 90.8% of all species (Lu, 2011). In fact, the distribution and diversity of plants in Kinmen have changed along with their development and decline throughout history. Chinese plants gradually dispersed to Kinmen when the island was connected to China during the fourth quarter of the Ice Age. However, after the Ice Age ended, the global sea level rose, and Kinmen and China were once again separated by water. For these reasons, the flora of Kinmen is now closely related to that of China. The main matrix of Kinmen is granite gneiss, which is distinct from the earth-rock matrix of Taiwan Island. Therefore, the plants in Kinmen are similar to those in China but quite different from those in Taiwan (Tseng, 2010). This led to some interesting questions: What are the influences of politics and this special geographical environment on the ethnobotany of Kinmen? Was plant use in Kinmen more influenced by Taiwan or China?

**FIGURE 1:**
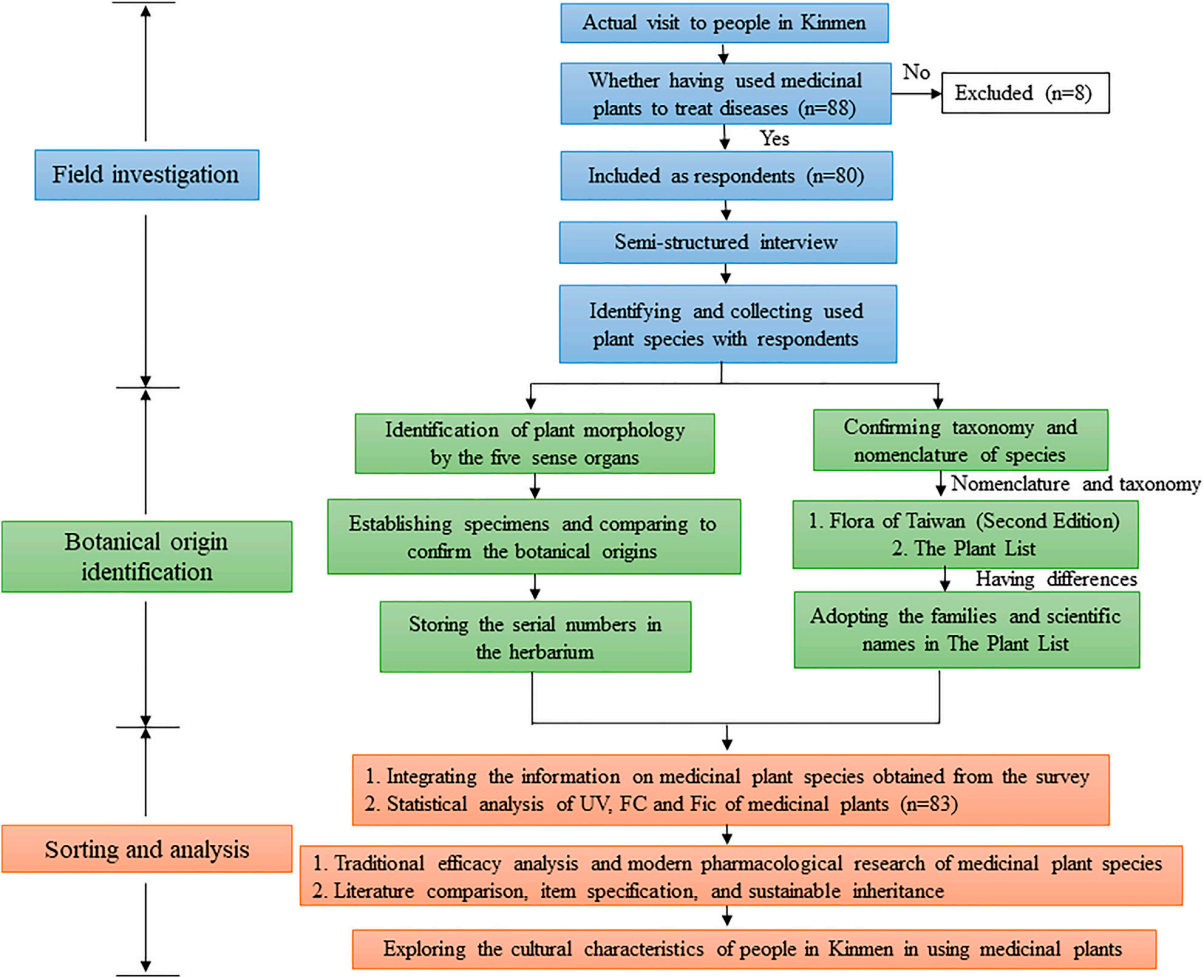
Research framework and process. Note: UV: use value, FC: frequency of citation, Fic: factor of informant consensus.

In the present study, we conducted an ethnobotanical study on the medicinal plants in Kinmen through a field investigation. We investigated how people in Kinmen use medicinal plants and recorded the traditional folk medicinal knowledge in the study area (Figure 1). This study is the first ethnobotanical study approved by the Institutional Review Board (IRB), which is of great significance. Furthermore, we analyzed the investigation results and systematically arranged them by integrating traditional pharmaceutical knowledge, plant taxonomy, and modern pharmacological analysis. The results were then compared with the representative literature on the ethnobotany of neighboring areas to analyze the cultural characteristics of medicinal plant usage in the Kinmen area. We expect this study to advance the ethnobotanical knowledge and future research of the medicinal plants in Kinmen.

## 2 Materials and Methods

### 2.1 Study Area

**FIGURE 2:**
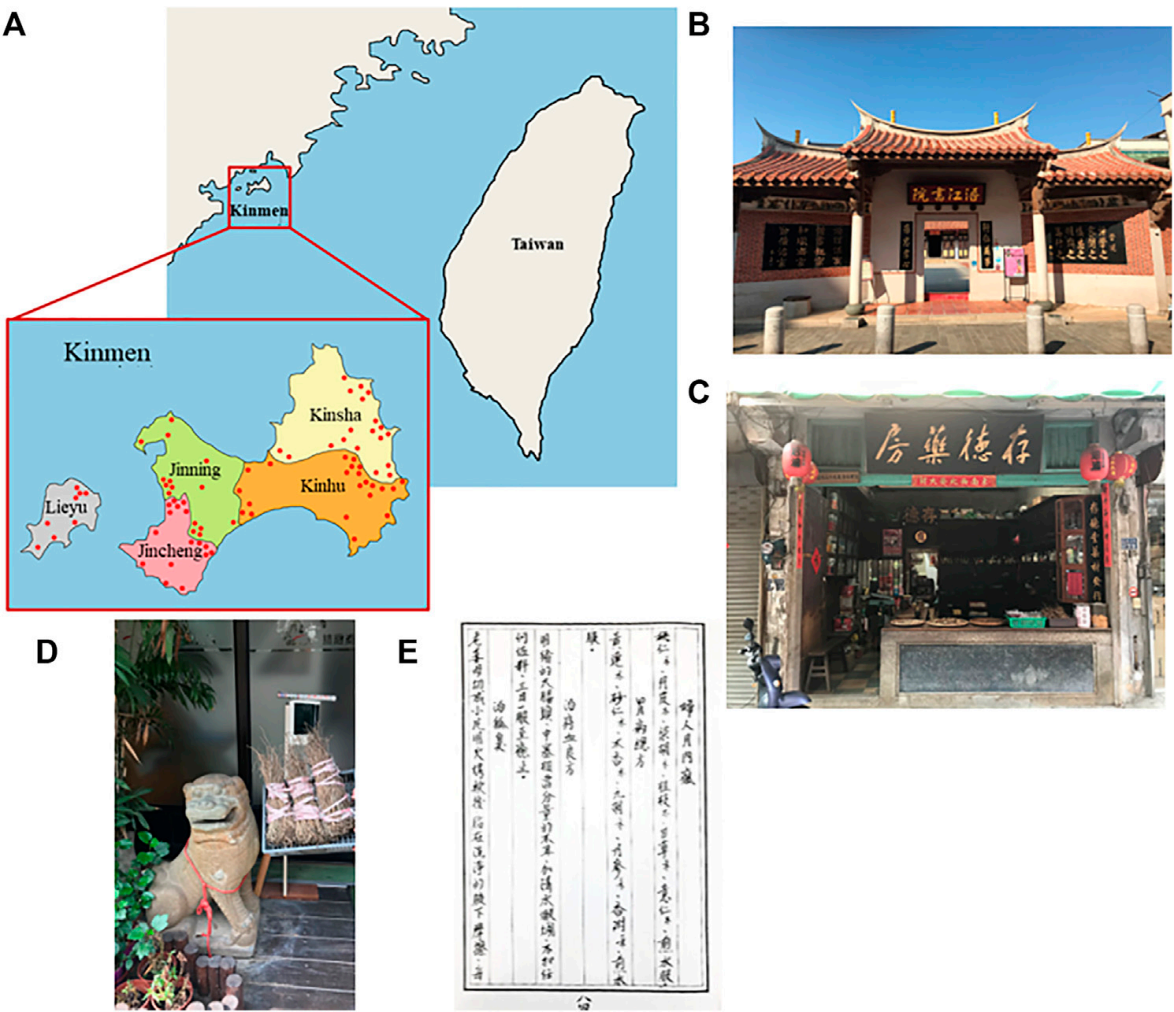
The Kinmen region *photos. **(A)** Map of the survey area; **(B)** Wujiang Academy (This academy used to be a school. Established in 1780, it is the last remaining academy out of four similar old Kinmen institutions.); **(C)** Cunde Chinese Pharmacy (established in 1832); **(D)** Kinmen Wind Lion God and *Glycine tomentella* Hayata (Yì Tiaó Gen); **(E)** hand-written traditional medicine prescriptions.

Kinmen is a mid-latitude island approximately 151.6 square kilometers in size. It is located on the southeast coast of Fujian Province, China, outside the Jiulong River Estuary (24°24′N–24°32′N, 116°28′–118°18′E), and is close to Xiamen Bay. It is 8 km away from the People’s Republic of China and 227 km away from Taiwan’s main island. Geologically, granite gneiss makes up its matrix, and the soil is mainly composed of brick-red clay soil, yellow-gray sandy soil, and bare rock areas (Lu, 2011). It is characterized by a subtropical monsoon climate with an average annual temperature of 20.9°C. The research area for this study was the Kinmen area (Figure 2), and the villages and towns that were included spanned Jincheng Town, Kinhu Town, Kinsha Town, Jinning Township, and Lieyu Township, which comprise the main population in Kinmen.

### 2.2 Ethnobotanical Investigation

This investigation was carried out from May 2019 to May 2020 (Supplementary Table S1). The research was approved by the IRB of China Medical University & Hospital Research Ethics Center (CRREC-108-055).

Intentional sampling was used for the survey since the sampling selection considered which research subjects could provide complete and sufficient data as well as direct help (Wu, 2014). The target subjects were local residents who had experience in using medicinal plants. The point of data saturation was defined as when the relevant data collected by the interviewer was continuous, but the content shared by the respondent became repetitive; once this occurs, the interviewers can stop recruiting cases (Walker, 2012). The sample number saturation referred to previous studies in the same field or topic, and most of them depended on the sampling method and the number of samples or questions in the questionnaire. Taking the questionnaire as an example, it was suggested that the number of samples should be 3–5 times or 5–10 times the number of questions (Comrey, 1988).

In the survey, a semi-structured interview was used to select the respondents through a local elderly individual. The target group included local people who had experience in using medicinal plants in Kinmen. The interview was divided into two parts. The first part collected basic information, such as the respondent’s name, age, gender, place of birth, and education level. The second part was a semi-structured questionnaire which consisted of six questions: (1) whether the respondent used any medicinal plants to treat or prevent diseases; (2) if yes, which diseases was the plant used to treat or prevent; (3) how the plants were used (including which parts, preparation methods, and use methods); (4) what are the sources of these plants; (5) how was this knowledge acquired; (6) what else should be added to this interview. The interview was conducted in Mandarin (Chinese) or Minnan (Hokkien/Taiwanese) dialects. During the interview, besides written records, audio recordings and photos were taken with the permission of the other party, and the respondents were asked to take us to the adjacent grassland, farmland, or mountain to identify the medicinal plants that they used and provide local colloquial names in Mandarin (Chinese) or Minnan (Hokkien/Taiwanese) dialects.

### 2.3 Identification of Botanical Origins

The medicinal plants used by the respondents were collected from the wild or plantings at the respondents’ homes. All species were identified according to morphology by Dr. Shyh-Shyun Huang (Associate Professor, Department of Pharmacy, China Medical University) and the authors of the present study. Plant specimens were collected and compared with the Specimens Database of Native Plants in Taiwan to confirm the species. Finally, the herbarium numbers (CMUK001-080) were stored in the herbarium of China Medical University.

In the process of botanical identification, the systematics, nomenclature, and taxonomy of the medicinal plant species used by the public were confirmed using the *Flora of Taiwan* (Second Edition) and *The Plant List*. The families and scientific names listed in The Plant List were used if there were differences in classification.

### 2.4 Data Collation and Analysis

During the investigation and interview, all of the collected data was compiled into a data table which included (1) families, (2) scientific names and specimen numbers, (3) plant names in the local colloquial language, (4) medicinal parts, (5) preparation methods, (6) use methods, (7) therapeutic uses, (8) plant use value (UV; the average of the total frequency of use of each medicinal plants), (9) frequency of citation (FC; the number of times a species has been used to treat a disease), (10) factor of informant consensus (Fic; an important index for treating diseases with medicinal plants), and (11) results of the network analysis.

For the above data ((1) and (2)) *The Plant List*, the second edition of *Flora of Taiwan* (Editorial Committee, 2003), and Taiwan Biodiversity Information Facility (http://taibif.tw/) were referred to. Items (3), (4), (5) and (6) are the data obtained from investigation. The therapeutic use (7) was conducted by arranging and comparing with *Flora of Kinmen* (Lu, 2011), the *Catalogue of Medicinal Plant Resources in Taiwan* (Executive Yuan Committee on Chinese Medicine and Pharmacy, 2003) and *Record of Fujian Materia Medica* (Fujian Academy of Medical Sciences, 1979). The activity of modern pharmacological research is anchored in the previous research results found in the online medical database (PubMed) established by the United States National Library of Medicine.

UV (8) is the average of the total frequency of use of medicinal plants, and is determined by calculating the total frequency of use of each plant species divided by the number of respondents.

$$\mathrm{UV}=\frac{\sum \mathrm{Ui}}{N}$$

where Ui is the number of use reports cited by each informant for a given species, and N is the total number of informants.

FC (9) is an important indicator of the frequency of use of a plant species for treating a disease, and it shows the number of times a species has been used to treat a disease.

Fic (10) is an important index for treating diseases with medicinal plants, and its value ranges from 0 to 1 (Heinrich et al., 1998; Andrade-Cetto and Heinrich, 2011). It is calculated as follows:

$$\mathrm{Fic}=\frac{\mathrm{Nur}-\mathrm{Nt}}{\mathrm{Nur}\mathrm{-1}}$$

where Nur refers to the number of times the respondents mention a medicinal plant used to treat a disease, and Nt refers to the number of respondents that mentioned a medicinal plant species for treating a disease. Diseases with a high Fic value indicate that local residents have shared opinions on which medicinal plants are used to treat these diseases. Conversely, diseases with a low Fic value indicate that the respondents have no consensus on using certain medicinal plants for treating these diseases.

With regard to the calculation and determination of the Fic value, first, we checked the Fic value of each disease; the greater the Fic value, the more useful the local residents believe the plants are for treating certain diseases. Thus, the diseases with higher Fic values were selected for further analysis. Second, the Nur of these diseases was checked; the greater the Nur, the more frequently local residents used these plants to treat these diseases, indicating that these diseases are likely to be “common local diseases.” Finally, according to Nt values, we could determine which plants are used to treat these diseases, among which we could determine the plants with higher FC values, which indicated that these plants are often used to treat these diseases and will have greater research value and development potential in the future.

Fic is used to classify the symptoms and diseases occurring locally according to the International Classification of Primary Care (ICPC) established by the World Organization of Family Doctors.

The Traditional Chinese Medicine Inheritance Support System (TCMISS) (11) was used for the network analysis (Wu et al., 2019; Wu et al., 2020). The survey results were determined by two parameters: degree of support and confidence score. The degree of support is defined as the frequency of collocation between plants and the confidence and conditional probability, which is defined as the probability of another species appearing at the same time when a certain medicinal plant species is known to be used. After systematic calculation, the network diagram showed the medicinal plants frequently used by the people in Kinmen and the categories of diseases that were treated most often.

## 3 Results and Discussion

### 3.1 Respondent Information

A total of 88 respondents were included in this survey. Eight respondents (four males and four females) had no experience in using medicinal plants to treat diseases. The remaining 80 people (35 males and 45 females) were completely interviewed, and their interviews were recorded in detail. Prior to the interviews, all of the respondents provided informed consent and signed a consent form. The respondents were from Jincheng Town (16.63% female), Kinhu Town (22.59% female), Kinsha Town (19.58% female), Jinning Township (15.33% female), and Lieyu Township (8.75% female). According to the survey, women could identify six species of medicinal plants on average, whereas men only knew four species on average. Therefore, compared to men, women were able to identify 1.5 times more medicinal plants. Among the participants, a male expert and a female (usually a housewife) couple were able to identify up to 18 medicinal species that have been used. Women were more familiar with the knowledge and use of medicinal plants than men were, which may be because women usually take care of their families in the traditional Southern Fujian society of Kinmen (Lu, 2014). Other studies have also shown that although men occupy a dominant position in the public professional field of plants, women still have more knowledge about the use of traditional medicinal plants than men (Eddouks et al., 2017). A survey in Taiwan found that men in the Miaoli Hakka ethnic group are more familiar with the use of medicinal plants than women are (Wu, 2007); therefore, in Southern Fujian culture society and Hakka culture, the awareness of plant usage is quite different between males and females.

**FIGURE 3:**
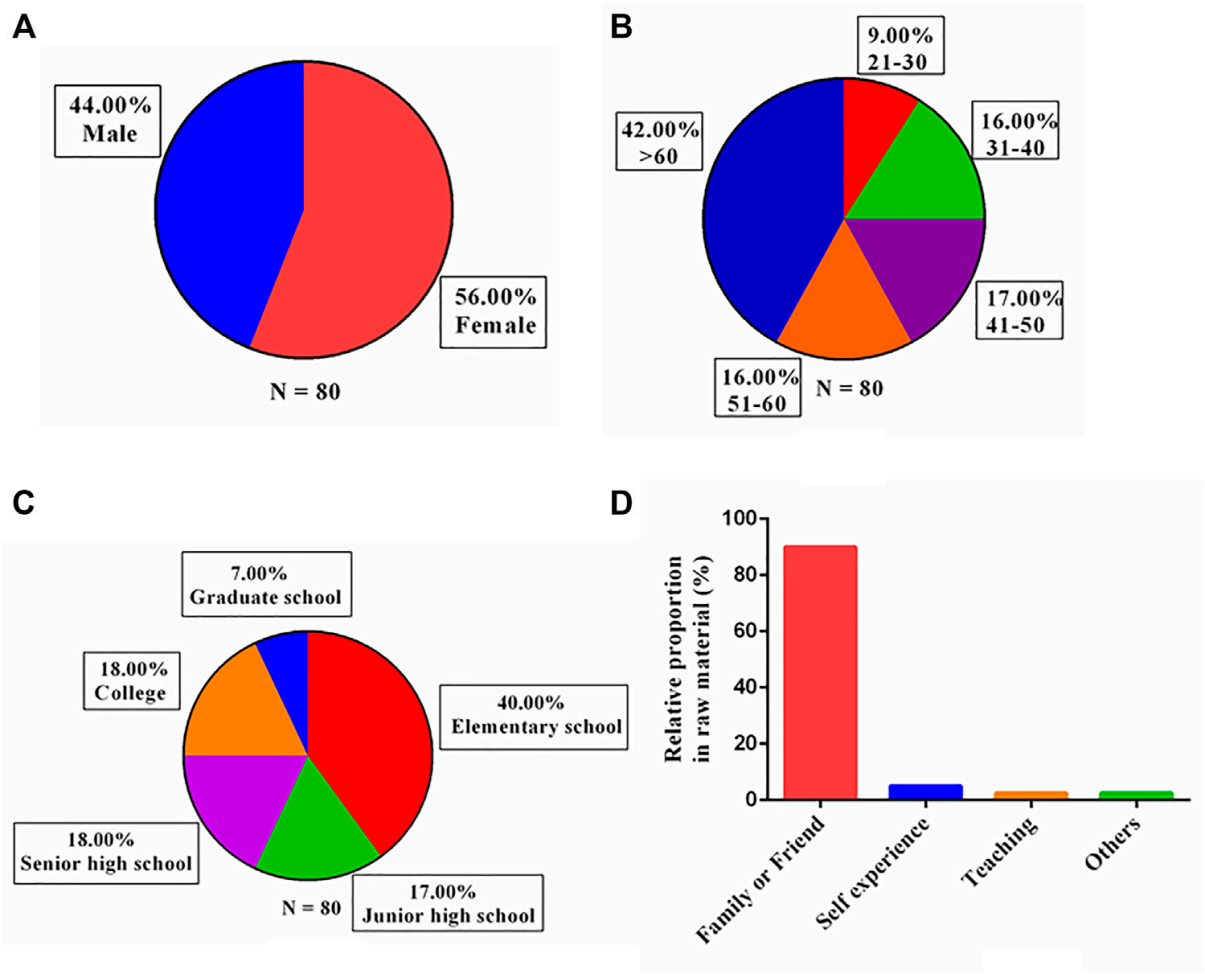
Respondent statistics. **(A)** Gender; **(B)** age (years); **(C)** education level; **(D)** knowledge sources (*N* = 90). Note: A respondent may have more than two knowledge sources, therefore *N* = 90.

As can be seen from the age distribution (Figure 3), 42% of the respondents (34 people) were over 60 years old, while only 9% (7 people) were young people (20–30 years old); eight respondents who were not included in the survey were under 30 years old. This ratio showed that different age groups have different knowledge about medicinal plants and that the age groups who used medicinal plants to treat diseases are mainly people over 50 years old (58%). Studies have shown that older people have more knowledge about the usage of medicinal plants (Wu, 2007; Bouasla and Bouasla, 2017; Miara et al., 2018; Lee et al., 2019). From the above results, it can be inferred that the elders and their second-generation descendants who had experienced a period of political and local warfare (A.D. 1945–1991) generally had more experience using medicinal plants to treat different diseases. This may be related to the history of the Kuningtou Battle (A.D. 1949) and Second Taiwan Strait Crisis (A.D. 1958–1979).

Most of the 80 respondents included in the study only had an elementary school (40%) or junior high school education (17%) (Figure 3), while the eight respondents who were not included were at or above the university education level. This phenomenon indicated that knowledge regarding medicinal plant usage is generally passed among people with only primary and secondary education. We speculated that this may be because the respondents with lower education levels are more interested in traditional medicine and have more frequent contact with plants. The respondents with higher education levels were less interested in traditional medicine and have been educated in modern society for a long time, and are less exposed to medicinal plants and relevant knowledge on traditional folk medicine. Similar results reported in other studies demonstrated that people with lower education levels, and even illiterate people, have more experience using medicinal plants than high-level intellectuals (Ahmad et al., 2017; Miara et al., 2018).

It is worth noting that with regard to the sources of knowledge for medicinal plant use, the proportion of inheritance through oral transmission was as high as 90% (Figure 3). In Kinmen, which is characterized by simple folk customs, sensitive and special political orientation, and military policies, information about herbal medicines are often hard to obtain because it is secret, and therefore, only given to children by elders, friends, or neighbors. This may be related to the fact that the second generation of descendants who experienced the period of political warfare (A.D. 1945–1991) had better knowledge of using medicinal plants.

### 3.2 Plant Species Used by People in Kinmen

#### 3.2.1 Botanical Diversity

A total of 83 species of medicinal plants were collected, including four fern species, and the collected species belonged to 48 families and 77 genera (Table 2; Supplementary Table S2).

**TABLE 2:** Statistics of the area where informants live in Kinmen.

Township	Male	Female
Jincheng Town	6	10
Kinhu Town	9	13
Kinsha Town	8	11
Jinning Township	8	5
Lieyu Township	2	6

**FIGURE 4:**
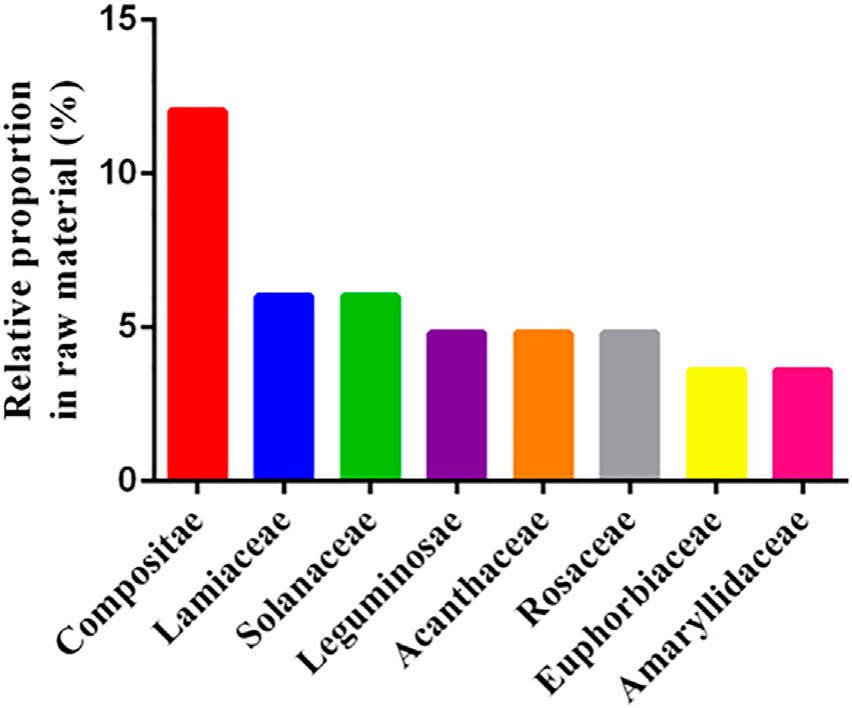
Statistics of common families (*N* = 83). Note: n was based on the number of collected species, not the number of families.

The highest number of species belonged to Compositae (12.04%), Lamiaceae (6.02%), and Solanaceae (6.02%), followed by Leguminosae (4.82%) and Acanthaceae (4.82%) (Figure 4). Therefore, Compositae species had the highest proportion of use. Similar results were obtained for both native plants of Taiwan (Yen, 2010) and folk plants in Kinmen (Lu, 2005). A possible reason for the high proportion of Compositae used in folk medicine is that these plants are abundant and include many species and varieties, which are closely related. According to the records in Volume 6 of *Flora of Taiwan*, 2nd Edition, more than 6,200 species of vascular plants have been identified in Taiwan, with Compositae being the third-largest vascular plant family, comprising about 242 species belonging to 85 genera. In addition, among the 820 vascular plants in the *Flora of Kinmen*, the family Compositae is the second largest plant family in Kinmen, with 44 genera and 77 species (this information can be found on page 14). Moreover, this family has the highest number of naturalized and invasive plants in the world (Chung and Peng, 2008), which is why Compositae species are easily available in the wild. According to our survey, most people in Kinmen obtain medicinal plants from the roadside and local sources, which is the reason why Compositae is the most widely used family.

#### 3.2.2 Frequently Used Species

**FIGURE 5:**
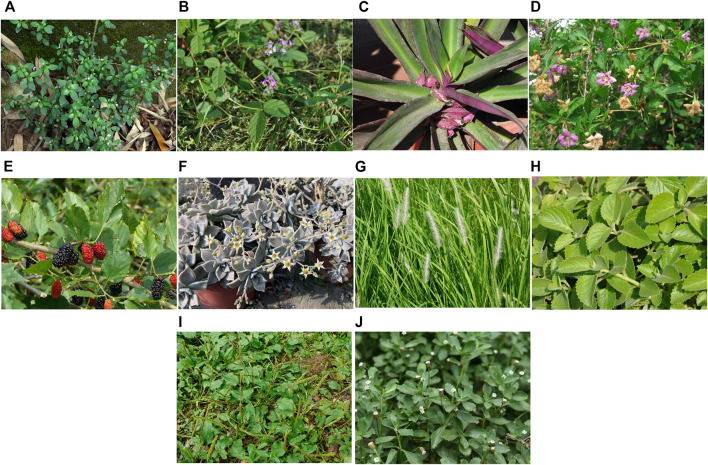
The medicinal plants most often used by people in Kinmen. **(A)** *Justicia procumbens* L.; **(B)** *Glycine tomentella* Hayata.; **(C)** *Tradescantia spathacea* Sw.; **(D)** *Lycium chinense* Mill.; **(E)** *Morus alba* L.; **(F)** *Graptopetalum paraguayense* (N.E.Br.) E. Walther; **(G)** *Imperata cylindrica* (L.) Raeusch.; **(H)** *Plectranthus amboinicus* (Lour.) Spreng.; **(I)** *Plantago asiatica* L.; **(J)** *Phyla nodiflora* (L.) Greene.

The plants most often used by people in Kinmen to treat diseases (UV) were *Justicia procumbens* L. (0.93), *Glycine tomentella* Hayata (0.45), and *Tradescantia spathacea* Sw. (0.41). These plants are often used by people in Kinmen and people from Taiwan or mainland China who settled in Kinmen. Other commonly used plants include *Lycium chinense* Mill. (0.4), *Morus alba* L. (0.34), *Graptopetalum paraguayense* (N.E.Br.) E. Walther (0.33), *Imperata cylindrica* (L.) Raeusch. (0.3), *Plectranthus amboinicus* (Lour.) Spreng. (0.28), *Plantago asiatica* L. (0.26), and *Phyla nodiflora* (L.) Greene (0.23) (Table 3, Figure 5).

**TABLE 3:** Basic data of typical medicinal plants (UV > 0.1) in Kinmen.

No	Family	Scientific name Voucher specimen number	Local name	Pars used	Preparation method	ΣUi	UV	FC	RFC	Ailments
1	Acanthaceae	*Justicia procumbens* L.	Yi Ạ Tsaǒ	Wp	Decoction/Oral	74	0.93	72	0.9	Allergic rhinitis, cold and cough, sore throat
2	Leguminosae	*Glycine tomentella* Hayata.	Yì Tiaó Gen	Ro	Soak or Decoction/Oral	36	0.45	33	0.41	Rheumatism, joint pain, contusion, reddish
3	Commelinaceae	*Tradescantia spathacea* Sw*.*	Hóng Jhú Yèh	Le	Decoction/Oral	33	0.41	20	0.25	Allergic rhinitis, cold, clear heat and resolve toxin
4	Solanaceae	*Lycium chinense* Mill.	Koǔ Ní Tsan Gan Goǔ Chǐ	Ro & Fr	Decoction/Oral	32	0.4	18	0.23	Fever, heat stroke, eye disease
5	Moraceae	*Morus alba* L	Suan Chaí	Fr	Raw or Decoction/Oral	27	0.34	20	0.25	Anemia
6	Crassulaceae	*Graptopetalum paraguayense* (N.E.Br.) E. Walther	Shíh Lián Hua	Le	Raw/Oral & Juice/Apply to skin	26	0.33	20	0.25	Liver diseases, sore throat antipruritic
7	Poaceae	*Imperata cylindrica* (L.) Raeusch	Mǎ Tsaǒ Gen	Wp	Decoction/Oral	24	0.3	20	0.25	Fever, cold, myalgia
8	Lamiaceae	*Plectranthus amboinicus* (Lour.) Spreng	Zuǒ Shoǔ Siang	Le	Crush/Apply to injuries	22	0.28	20	0.25	Contusion, joint pain, reddish
9	Plantaginaceae	*Plantago asiatica* L	Wǔ Jin Tsaǒ	Wp	Decoction/Oral	21	0.26	16	0.2	Flatulence, cough, bronchitis
10	Verbenaceae	*Phyla nodiflora* (L.) Greene	Ya Shé Hóng	Le	Crush/Oral	18	0.22	13	0.16	Sore throat
11	Euphorbiaceae	*Jatropha curcas* L	Baí Tǔ Pí	St&Le	Juice/Apply to injuries	16	0.2	13	0.16	Recurrent aphthous stomatitis, herpes
12	Lamiaceae	*spicata* L	Bò Hé	Le	Soak or Decoction/Oral	12	0.15	9	0.11	Headache, Sore throat
13	Leguminosae	*Senna tora* (L.) Roxb	Jyuéh Míng	Se	Decoction/Oral	12	0.15	9	0.11	Promoting urination, eye diseases
14	Cucurbitaceae	*Momordica charantia* L	Shan Kǔ Gua	Fr	Decoction/Oral	12	0.15	8	0.1	Clear heat and resolve toxin, diabetes
15	Compositae	*Bidens pilosa* L	Siaò Chá Moǔ	Wp	Decoction/Oral	12	0.15	7	0.09	Clear heat and resolve toxin, diabetes, diarrhea
16	Compositae	*Cirsium japonicum* (Thunb.) Fisch. ex DC.	Ji Jiaǒ Jì	Wp	Decoction/Oral	12	0.15	7	0.09	Liver diseases, diabetes
17	Compositae	*Bidens bipinnata* L	Shén Jhen Tsaǒ	Wp	Decoction/Oral	10	0.13	6	0.08	Clear heat and resolve toxin, gout
18	Amaryllidaceae	*Crinum asiaticum* L	Yǐn Shueǐ Jiao	Le	Raw/Fomentation	8	0.1	8	0.1	Skin redness
19	Basellaceae	*Anredera cordifolia* (Ten.) Steenis	Chuan Chi/Yún Nán Baí Yaò	Le	Crush/Apply to injuries	8	0.1	8	0.1	Hemorrhage
20	Compositae	*Chrysanthemum morifolium* Ramat.	Jyú Hua	Le	Decoction/Oral	8	0.1	6	0.08	Eye disease, dizziness
21	Compositae	*Crossostephium chinense* (A. Gray ex L.) Makino	Haǐ Fú Róng	Wp	Decoction/Oral	8	0.1	6	0.08	Rheumatism, joint pain
22	Dioscoreaceae	*Dioscorea alata* L.	Shan Yaò	St	Decoction/Oral	8	0.1	6	0.08	Digestion, stomach disorder
23	Lamiaceae	*Ocimum basilicum* L.	Jioǔ Tséng Tǎ	Le & Ro	Le: Fry/Chew Ro: Cook with meat/Oral	8	0.1	6	0.08	Blood purifier
24	Leguminosae	*Senna occidentalis (L.)* Link	Shan Kafei	Se	Decoction/Oral	8	0.1	6	0.08	Eye diseases

Parts used: Le, leaves; St, stems; Ro, roots; Fr, fruits; Wp, whole plant; Se, seeds.

The whole plant of *Justicia procumbens*, locally known as Yi Ạ Tsaǒ, is boiled and taken orally to treat colds, sore throat, and allergic rhinitis. Its traditional effects include clearing heat, removing toxicity, promoting urination, promoting blood circulation, and relieving pain, as well as treating cold, fever, dysentery, jaundice, and traumatic injuries (Executive Yuan Committee on Chinese Medicine and Pharmacy, 2003). In Fujian, China, it is used to clear heat, remove toxicity and fluid retention, promote urination and blood circulation, and relieve pain (Fujian Academy of Medical Sciences, 1979). The *Flora of Kinmen* records that the whole plant is used as medicine to treat sore throat and lower back pain. The known pharmacological activities of this species are antioxidant (Charoenchai et al., 2010), antiviral (Xu et al., 2019), antibacterial (Zhang et al., 2007), and anticancer activity (Day et al., 2002; Wang et al., 2015). It is worth noting that this plant is widely used by people in Kinmen because of its efficacy in clearing heat and removing toxicity, as documented in different literature and studies.

*Glycine tomentella* (broad-leaved soybean) is a medicinal plant native to Kinmen (Lu, 2011). It is used to treat musculoskeletal problems, particularly a condition locally called Yì Tiaó Gen. The root quality of plants cultivated in Kinmen is high because of the windy climate, fertile red soil, and water quality. Therefore, in Kinmen, this plant is thought to be superior to Korean ginseng, and its medicinal effect is well-known. Studies have shown that broad-leaved soybean has antioxidant (Wang et al., 2012) and low-density lipoprotein-reducing properties (Chen et al., 2005), and its analgesic and anti-inflammatory activities have been supported in several published studies (Chen et al., 2005; Yen et al., 2010; Chuang and Pan, 2011; Wang et al., 2012; Wu et al., 2019). It is also a well-known medicinal plant in neighboring countries, especially China and Taiwan, and is often used to treat rheumatic joint pain and traumatic bone and tendon injuries (Executive Yuan Committee on Chinese Medicine and Pharmacy, 2003; Fujian Academy of Medical Sciences, 1979).

In Kinmen, the species *Tradescantia spathacea* is called Hóng Jhú Yèh. It is collected fresh, boiled, and taken orally. Its effects are: clearing heat, relieving fever, and treating the common cold. Studies have confirmed its antioxidant and anti-inflammatory activities (Cambie and Ferguson, 2003). This plant is not mentioned in the studies of *Flora of Kinmen*, *Ethnobotany in Kinmen*, or the *Record of Fujian Materia Medica*, but it is widely used in Taiwan to treat blood stasis, pneumonia, cough, and traumatic injuries (Executive Yuan Committee on Chinese Medicine and Pharmacy, 2003).

In the present study, we speculated that local people often use the heat-clearing and detoxifying effects of the three species described in this section to treat respiratory problems and fever. When we discussed the reasons for their common usage, we found that local residents mainly worked in agriculture, industry, and fisheries and are prone to cold and heatstroke, which is why there is an urgent need to reduce internal heat (Lu, 2005). Many studies have speculated that the mechanism of heat-clearing and annealing may be related to the plant antioxidant capacity (Huang et al., 2020). Therefore, Compositae and Lamiaceae plants with antioxidant effects correspond to the commonly used families in the study area.

#### 3.2.3 Sources of Plants

Among the 83 medicinal species used by people in Kinmen, 67 species (80%) were collected from local roadsides, fields, and mountains, and the remaining 16 species (20%) were obtained from the Agricultural Research Institute. This situation is very different than that in Taiwan, where herbs are sold in drugstores (Chang, 2004). According to our field investigation, there is no such marketplace in the study area, which is why compared to Taiwanese people, people in Kinmen seem to rely more on wild plants.

Our investigation found that some people used the medicinal plants incorrectly, for example, they used *Heliotropium strigosum* Willd. instead of *Oldenlandia diffusa* (Willd.) Roxb. This may be because both of them have the effect of clearing heat and removing toxicity after the whole plant is boiled, and their appearance and shape are similar. People may also mistake *Aristolochia cucurbitifolia* Hayata for *Aristolochia kaempferi* Willd. The use of this plant species is unique in Taiwan, because it is mashed and applied externally, which has the effect of resolving swelling and pain and treating sores and carbuncles. On the other hand, in the process of identifying the origin, one plant species is widely used by the people of Kinmen, but they only know its local colloquial name Mǎ Tsaǒ Gen, and no one knew its scientific name. After Dr. Shyh-Shyun Huang identified this species using crude field methods, it was preliminarily determined to be a Gramineae species, and its origin could not be confirmed after specimen comparison. Therefore, the plant origin was identified through DNA barcoding, a method of analyzing and identifying samples according to standard DNA sequences. The genetic similarity analysis of the sample sequences was carried out by the National Center for Biotechnology Information (NCBI) in the United States using sequence alignment with the tested object (de Vere et al., 2015; Li et al., 2015). The sample sequence of the studied species and that of *Imperata cylindrica* were over 97% similar after confirming the origin of this species repeatedly. This plant was identified as *Imperata cylindrica* (L.) Raeusch., and its DNA sequence was uploaded to NCBI (accession number: PRJNA635266) to establish its sample sequence.

### 3.3 Medicinal Plant Parts and Methods of Use

**FIGURE 6:**
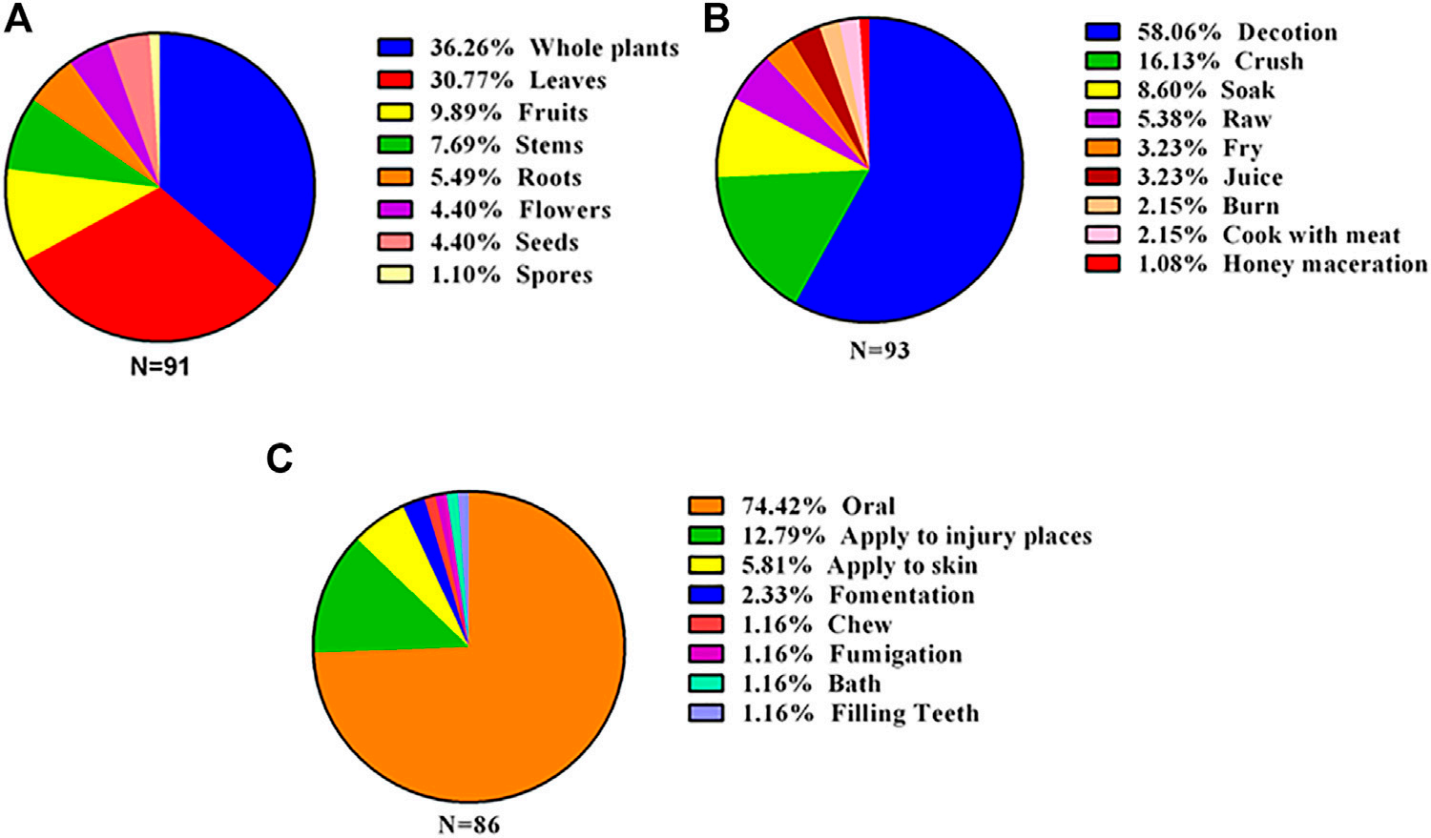
Statistics on the use of medicinal plants in Kinmen. **(A)** Medicinal parts (*N* = 91): if there were more than two kinds of medicinal parts of the same plant, it was counted as 2, etc.; **(B)** preparation methods (*N* = 93): if the same plant had more than two preparation methods, it was counted as 2, etc.; **(C)** usage method (*N* = 86): if the same plant had more than two usage methods, it was counted as 2, etc. Note: soak (soaking, including making tea); juice (using plant juice).

In Kinmen, the parts of medicinal plants utilized by people are as follows; whole plants are used (36.26%) in most cases, followed by leaves (30.77%) and fruits (9.89%) (Figure 6). This may be related to the lack of information and medical knowledge in the past. Leaves may be commonly used because they are easier to obtain than other plant parts and less likely to negatively affect plant growth (Mesfin et al., 2013; Miara et al., 2018; Lee et al., 2019). In addition, we found that people in Kinmen are similar to residents of Taiwan and Fujian; they often use different parts of the same plant to achieve different effects. For example, the fruit of mulberry (*Morus alba* L.) has the effect of nourishing yin and enriching blood, but its leaves have a diuretic effect. Furthermore, its fruits can be used for improving eyesight and curing dry eyes, but after drying and boiling them with the roots of *Lycium chinense*, they can be used for annealing and curing heatstroke. In Kinmen, Taiwan, and even Fujian, China, *Lycium barbarum* is used for nourishing the liver, improving eyesight, clearing heat, and annealing (Fujian Academy of Medical Sciences, 1979; Lu, 2011; Executive Yuan Committee on Chinese Medicine and Pharmacy, 2003), and its antioxidant activity has been confirmed by many studies (Vulic et al., 2016; Shan et al., 2019).

Regarding the herb preparation methods (Figure 6), there were seven different modes of treatment, among which decoction (58.06%) was the most common. Decoction included fresh decocting (42.76%) and decocting after drying (15.3%). The other preparation methods were crushing (16.13%), soaking (8.60%), using raw herbs (5.38%), frying (3.23%), juicing (3.23%), burning (2.15%), cooking with meat (2.15%), and honey maceration (1.08%). Most residents used fresh herbs or prepared them by fresh cooking, mashing, soaking, and juicing. Furthermore, we found that people in Kinmen have different preparation methods for the same plant to treat different diseases. When using *Oxalis debilis* var. *corymbosa* (DC.) Lourteig, taking an herb bath in hot water is used to treat children’s skin rash, while its bulbs can be boiled fresh and used to promote urination and eliminate edema. In addition, the whole plant can be mashed and added to a mixture of water and salt and is drunk to treat a sore throat. This information is very similar to that reported in the document *Knowledge of Herbal Medicines in Kinmen in 1981*, indicating that local people still use medicinal plants according to ancient laws and methods described in ancient books.

Regarding the methods of use (Figure 6), there are seven different ways to administer these herbs, and the most commonly used methods of people in Kinmen are as follows: oral ingestion (74.42%), topical application on injuries (12.79%), skin application (5.81%), fomentation (2.33%), chewing (1.16%), bath (1.16%), fumigation (1.16%), and filling teeth (1.16%). Consistent with most research results, oral ingestion is the most commonly used way of taking medicine (Yemele et al., 2015; Ahmad et al., 2017; Ribiero et al., 2017; Miara et al., 2018; Lee et al., 2019).

### 3.4 Data Collation and Analysis

#### 3.4.1 Therapeutic Application of the Recorded Medicinal Plants

From the aspect of therapeutic application, the Nur value shows which diseases were often treated with plants by the people in Kinmen. According to our survey data, most (22%) of the reported species were used to treat respiratory symptoms. The book *Investigation of Ethnobotany in Kinmen* also indicated that in Kinmen, most plant species are used to treat the common cold (Lu, 2005). In our survey, respiratory disease treatments were the most often mentioned by the respondents, and they had the highest number of citations (Nur = 136), with 18 plant species being used for these treatments (Nt = 18). This resulted in a high Fic value (0.87) (Table 4), with *J. procumbens* (FC = 72) and *T. spathacea* (FC = 24) being the most commonly used species by local people to treat respiratory diseases. According to the existing literature reports, respiratory ailments are an important disease category in Kinmen, and allergic rhinitis is the most common disease (Tsai, 2003; Hsu, 2007). *Ambrosia artemisiifolia L. Ambrosia artemisiifolia* In addition to pigweed, the Santa Maria feverfew (*Parthenium hysterophorus* L.) is another allergenic exotic plant from America that has spread all over Kinmen. A previous study found that about half of Kinmen patients with allergic rhinitis were allergic to the Santa Maria feverfew (Tsai, 2003). According to our on-the-spot investigation, as there are no factories in Kinmen, local allergic factors caused by industrial air pollution should not be a contributing factor.

**TABLE 4:** Respondents’ consensus on the use of medicinal plants.

No	Category	Use report (Nur)	Number of taxa (Nt)	Fic (factor of informant consensus)
1	Musculoskeletal diseases	63	5	0.94
2	Eye diseases	31	4	0.90
3	Respiratory diseases	136	18	0.87
4	Diseases related to blood, blood-forming organs, lymph, spleen	50	10	0.82
5	General and unspecified diseases	102	21	0.80
6	Digestive diseases	58	16	0.74
7	Urological diseases	18	7	0.65
8	Female genital system and breast diseases	3	2	0.5
9	Endocrine, metabolic, and nutritional diseases	18	10	0.47
10	Psychological diseases	4	3	0.33
11	Cardiovascular diseases	1	1	0
12	Male genital system diseases	1	1	0

Furthermore, we found that the disease category of musculoskeletal ailments had the highest Fic value (Fic = 0.94) (Table 4) and was mostly treated by two plant species. The first is *Glycine tomentella* (FC = 35), which is a native medicinal plant whose roots are the most commonly used in Kinmen. The Kinmen Agricultural Research Institute encourages farmers to plant this species, making it an important and commonly used local species. The other species is *Plectranthus amboinicus* (FC = 20), proven to have good analgesic and anti-inflammatory activities by modern pharmacological research (Gurgel et al., 2009; Chiu et al., 2012). As it is easy to plant, it is widely used by the local people.

The residents of Kinmen reached a strong consensus (Fic = 0.90) (Table 4) regarding the treatment of eye disease. They use *Lycium chinense* (FC = 12), *Senna tora* (L.) Roxb. (FC = 9), and *Chrysanthemum morifolium* Ramat. (FC = 5) to improve eyesight and treat dry eyes. These plants are known for their eyesight-improving activities. According to traditional Chinese medicine, *Chrysanthemum* has the effect of dispelling wind and heat, and cassia seeds clear the liver and improve eyesight. These plants also have the functions of nourishing the liver and blood, and can be used to relieve fatigue (Chen, 2020).

In the present study, we also found that men in Kinmen generally use several liver-protecting plants found in the area for treating liver diseases, namely: *Graptopetalum paraguayense*, *Vernonia amygdalina* Delile, *Cirsium japonicum* (Thunb.) Fisch. ex DC., and *Tithonia diversifolia* (Hemsl.) A. Gray (Nt = 4). Furthermore, plants used to treat liver diseases (Nur = 33) also had a high Fic value of 0.9. We speculated that liver disease among men may be caused by the culture of drinking sorghum in Kinmen. In addition, during the war preparation period, the local residents could only eat “war preparation rice” (a type of long-grain rice with high aflatoxin levels) and moldy food, which led to liver problems in the elderly people of Kinmen (Kinmen National Park Headquarters, 2005).

#### 3.4.2 Item Specification of the Recorded Medicinal Plants

Traditional Chinese medical theory holds that many Chinese herbal medicines can be used as both medicines for treating diseases and as suitable food sources (Hung, 2005). Therefore, when studying the usage of the 83 medicinal plant species reported in our investigation of the literature, we found that 32 species are included in the list of raw materials used for food preparation, and seven plant species mentioned as Chinese medicinal materials can also be used for food. Unfortunately, common species (UV > 0.2) such as *Justicia procumbens* (0.93), *Tradescantia spathacea* (0.41), *Graptopetalum paraguayense* (0.33), *Plectranthus amboinicus* (0.28), and *Phyla nodiflora* (0.23) were not included in the list of raw materials used for food preparation. Furthermore, we found that 19 types of products made from the plants recorded in the present study are included in the *Taiwan Herbal Pharmacopeia*, and that 42 species recorded in the present study are not included in any of the three editions, accounting for more than half of the total number of recorded medicinal plant species in Kinmen. Therefore, these 42 species cannot be regulated at present, regardless of medicine and food standards. This topic may need to be discussed in more detail in the future, including making adjustments in managing raw materials used as both medicine and food.

#### 3.4.3 Sustainability of the Recorded Medicinal Plants

Ecological conservation has always been a very important issue. In the present study, the plants most commonly used by the residents were often the common and easily available plants, and most of them were collected in the wild. Therefore, awareness regarding the conservation status of these plants and their ecological environment is extremely important. A total of 83 medicinal plant species were collected in the Kinmen area, of which 34 species were recorded in the 2017 Red List of Vascular Plants in Taiwan. Among them, one species, *Myoporum bontioides* (Siebold & Zucc.) A. Gray, is endangered (EN); two species, *Crossostephium chinense* (ex L.) Makin, and *Solanum incanum* L. are vulnerable (VU); one species, *Potentilla discolor* Bunge, is near threatened (NT); 13 species are of least concern (LC); one species is data deficient (DD); and 16 species have not been assessed (NA). It is particularly important to achieve a balance between their conservation and use.

#### 3.4.4 Newly Discovered Species and Uses

Among the 83 species collected during our survey, 50 species (60.24%) were recorded in the *Flora of Kinmen,* and 14 had different reported effects from those recorded in the literature. For example, the seeds of *Senna occidentalis* (L.) Link are boiled by local people and used to clear the liver, improve eyesight, and promote urination, while the literature records state that they are used for “invigorating stomach and improving intestines.” Furthermore, we recorded that the whole plants of *Mimosa pudica* L. are used for relieving cough, eliminating phlegm, and treating the common cold after being boiled fresh, while the literature records state that it is used for “resolving inflammation, relieving pain, and removing edema.”

Out of the 83 species, 78 (93.98%) were listed in *The Catalogue of Medicinal Plant Resources in Taiwan,* and 18 were reported to have different functions from recorded literature. For example, people in Kinmen use the leaves of *Plectranthus amboinicus* mashed with sesame oil for external application to treat traumatic injuries, blood stasis, and swelling, while in the literature, this species is used for “aromatizing turbidity, appetizing and relieving vomiting, and relieving summer heat.” Furthermore, we recorded that the leaves of *Psidium guajava* L. are used to make tea which is drunk to lower blood glucose, while in the literature, this species is used for “astringing intestines, stopping bleeding, and dispelling parasites.”

Among the 83 species recorded here, 54 (65.06%) were recorded in the *Record of Fujian Materia Medica,* and 19 were reported to have different uses from the recorded literature. The flowers of *Epiphyllum oxypetalum* (DC.) Haw. were reported to be used dried and soaked in honey to treat sore throat, while the literature stated that it is used for “cooling blood and stopping bleeding.” The stems of *Solanum incanum* were reported to be dried and boiled to treat hepatitis and liver cirrhosis, while the literature records it is used for “promoting blood circulation, dispelling blood stasis, relieving pain, and anesthesia.” These findings verify the argument that “each locale has its own way of supporting its own inhabitants” and that nourishment and medicines are naturally available to each locality based on their local conditions and environment.

In the present study, 18 species of medicinal plants (21.70%) were observed in Kinmen for the first time (Table 5). People in Kinmen use the whole plant of *Portulaca oleracea* L. to treat hypertension, and the whole plant of *Cirsium japonicum* for liver protection, while the stinging cones of *Krameria prostrata* Brandegee are used for. The fruits can be used to treat sore throat, the whole plants of *Leucas chinensis* (Retz.) Sm. are boiled to treat diarrhea, and hot compresses with the leaves of *Raphanus raphanistrum* subsp. *sativus* (L.) Domin. are used to treat chilblain, which seems to be a unique usage of this species in the Kinmen area.

**TABLE 5:** Newly discovered species and uses in Kinmen.

Scientific name (Chinese name)	Utilized part	Usage not yet recorded in the literature
*Vernonia amygdalina* Delile (南非葉)^a^	Leaves	Treating liver disease and tonifying the kidneys
*Plectranthus amboinicus* (Lour.) Spreng. (到手香)	Leaves	Traumatic injuries, blood stasis, and swelling
*Agrimonia pilosa* Ledeb. (龍芽草)	Whole plant	Tonifying qi
*Pentacoelium bontioides* Siebold & Zucc. (苦檻藍)	Whole plant	Anti-inflammatory
*Bryophyllum delagoense* (Eckl. & Zeyh.) Druce (洋吊鐘)^a^	Whole plant	Hemorrhagic dengue fever
*Hibiscus sabdariffa* L. (洛神葵)	Calyx	Increasing metabolism and eliminating edema
*Orostachys fimbriata* (Turcz.) A. Berger (瓦松)^a^	Whole plant	Red and swollen wounds
*Osmanthus fragrans* Lour. (桂花)	Flowers	Treating halitosis and eliminating phlegm
*Heliotropium strigosum* Willd. (細葉天芥菜)^a^	Whole plant	Clearing heat and removing toxicity; preventing cancer
*Raphanus raphanistrum* L. (蘿蔔)	Leaves	Treating chilblain
*Leucas chinensis* (Retz.) Sm. (白花草)	Whole plant	Antidiarrheal
*Euphorbia thymifolia* L. (小飛揚草)	Whole plant	Eliminating flatulence and softening stool
*Scutellaria barbata* D. Don (半枝蓮)	Whole plant	Hepatitis
*Wedelia prostrata* Hemsl. (單花蟛蜞菊)	Whole plant	Removing blood stasis and swelling
*Krameria prostrata* Brandegee (刺球果)	Fruit	Sore throat
*Portulaca oleracea* L. (馬齒莧)	Leaves	Hypertension
*Cirsium japonicum* (Thunb.) Fisch. ex DC. (南國小薊)	Whole plant	Protecting liver
*Toona sinensis* (Juss.) M. Roem. (香椿)	Leaves	Promoting urination

Note

a signifies new medicine types in Kinmen.

In the literature of related medicinal plants in Kinmen, four medicinal plants are not included: *Vernonia amygdalina*, *Bryophyllum delagoense* (Eckl. & Zeyh.) Druce, *Orostachys fimbriata* (Turcz.) A. Berger, and *Heliotropium strigosum*. It has been pointed out that the leaves of *V. amygdalina* have anti-cancer (Johnson et al., 2017) and anti-malarial activities (Adia et al., 2014), while the medicinal effects of the other three plants have not been recorded in literature or modern pharmacological studies. However, the use and efficacy of 39 species recorded in the present study (46.99%) are consistent with those recorded in the *Catalogue of Medicinal Plant Resources in Taiwan* and *Record of Fujian Materia Medica*. Therefore, medicinal plant therapy in the Kinmen area is strongly influenced by Taiwan and the Fujian area of China.

The results of this study showed that the flora of Kinmen is similar to China but is quite different from Taiwan Island (Lu, 2011). Furthermore, applications of medicinal plants in China are similar to those in Taiwan (93.98%); however, medicinal plant usage in Kinmen is not similar to those in Fujian nor mainland China despite its geographical and environmental proximity. Importantly, nearly half of the medicinal plants used by people in Kinmen (46.99%) are shared with those used by people in Taiwan and Fujian. Although Kinmen is geographically separated from Fujian, China, by the Taiwan Strait, they share some of the same phytotherapies.

## 4 Conclusion

This study is the first IRB-approved study that recorded the practical use of medicinal plants by the people of Kinmen through interviews. As a result, 83 species of medicinal plants were collected, belonging to 48 families and 77 genera. Many commonly used plants, such as *Justicia procumbens* L., *Glycine tomentella* Hayata, and *Tradescantia spathacea* Sw., were readily available, or the farmers of Kinmen were encouraged to plant them on the roadsides. These plants are well-known and common in the local environment. Eighteen new medicinal uses that had not been recorded in the literature on Kinmen were also discovered. The therapeutic effects of some of these plants have been confirmed by research, but the pharmacological effects and safety of others have not yet been established. The four newly discovered medicinal species in the Kinmen area were *Vernonia amygdalina* Delile, *Bryophyllum delagoense* (Eckl. & Zeyh.) Druce, *Orostachys fimbriata* (Turcz.) A. Berger, and *Heliotropium strigosum* Willd. These species should be endemic or exotic species in the studied ecological environment. In addition, endangered species included *Myoporum bontioides*, *Crossostephium chinense* (A. Gray ex L.) Makin, *Solanum incanum* L., and *Potentilla discolor* Bunge. From the standpoint of sustainable conservation, these plants should be cultivated artificially as much as possible. The present study will provide a basis for phytochemical and pharmaceutical research and the conservation of these plant species.

Although the flora of Kinmen is similar to those in China and is quite different from those in Taiwan Island, some of the medicinal species used in Kinmen are used in Taiwan as well, but are not used in Fujian, despite their geographical and environmental proximity. It is important that the people of Kinmen share the applied use of medicinal plants with people in Taiwan and Fujian, and there are many things in common in their use of plants, including common medicinal families, use of particular plant parts, preparation methods, and usage methods. The observed difference is that people in Kinmen rely more on the use of wild plants; they have no cultural habit or place for purchasing herbs, which is why most people collect them from the roadside and mountains.

Kinmen has been deeply influenced by population migration and war because of its special geographical location, making the lifestyle of Kinmen people unique; nevertheless, the knowledge exchange with Taiwan and Fujian has lasted for centuries. In addition, in the past, people from China and Taiwan could only access Kinmen by transiting through Hong Kong for entry and exit. However, after the opening of the Mini Three Links, Kinmen became an important bridge between Taiwan and China. This made contact and communication between Kinmen and cross-strait areas more frequent. Knowledge of plants in Taiwan and Fujian or medicinal knowledge recorded in literature were passed on to people in Kinmen through oral transmission and parental instruction from people across the Taiwan Straits. Therefore, changes in the environment and time greatly affected the relationships between humans and plants (Plant science’s human factor, 2015; Pardo-de-Santayana and Macia, 2015), thus connecting the ethnobotanical knowledge of Kinmen, Taiwan, and Fujian in China.

## Data Availability

The original contributions presented in the study are included in the article/Supplementary Material, further inquiries can be directed to the corresponding author.

## References

[B1] Adia M. M. Anywar G. Byamukama R. Kamatenesi-Mugisha M. Sekagya Y. Kakudidi E. K. (2014). Medicinal Plants Used in Malaria Treatment by Prometra Herbalists in Uganda. J. Ethnopharmacol 155, 580–588. 10.1016/j.jep.2014.05.060 24928824

[B2] Ahmad K. S. Hamid A. Nawaz F. Hameed M. Ahmad F. Deng J. (2017). Ethnopharmacological Studies of Indigenous Plants in Kel Village, Neelum Valley, Azad Kashmir, Pakistan. J. Ethnobiol. Ethnomed 13, 68. 10.1186/s13002-017-0196-1 29191238 PMC5709976

[B3] Andrade-Cetto A. Heinrich M. (2011). From the Field into the Lab: Useful Approaches to Selecting Species Based on Local Knowledge. Front. Pharmacol. 2, 20. 10.3389/fphar.2011.00020 21954385 PMC3108584

[B5] Bouasla A. Bouasla I. (2017). Ethnobotanical Survey of Medicinal Plants in Northeastern of Algeria. Phytomedicine 36, 68–81. 10.1016/j.phymed.2017.09.007 29157830

[B6] Cambie R. C. Ferguson L. R. (2003). Potential Functional Foods in the Traditional Maori Diet. Mutat. Res. 523-4, 109–117. 10.1016/S0027-5107(02)00344-5 12628508

[B8] Charoenchai P. Vajrodaya S. Somprasong W. Mahidol C. Ruchirawat S. Kittakoop P. (2010). Part 1: Antiplasmodial, Cytotoxic, Radical Scavenging and Antioxidant Activities of Thai Plants in the Family Acanthaceae - Part 1. Planta Med. 76, 1940–1943. 10.1055/s-0030-1250045 20556707

[B9] Chen C. C. (2017). Overview of Kinmen Studies. Taiwan. Taiwan: Tung Hua Book Co. Ltd.

[B10] Chen D. H. (2020). How to Use Traditional Chinese Medicine Correctly. New York: Wordshop Publications Inc.

[B12] Chen T. Y. Shiao M. S. Pan B. S. (2005). Inhibition of 12- and 15-lipoxygenase Activities and protection of Human and tilapia Low Density Lipoprotein Oxidation by I-Tiao-Gung (Glycine Tomentella). Lipids 40, 1171–1177. 10.1007/s11745-005-1482-1 16459930

[B13] Chiang P. H. (2004). Investigation of the Current Status of Green Herb Stores in Taiwan. Taiwan: China Medical University.

[B14] Chiu Y. J. Huang T-H. Chiu C-S. Lu T-C. Chen Y-W. Peng W-H. (2012). Analgesic and Antiinflammatory Activities of the Aqueous Extract from Plectranthus Amboinicus (Lour.) Spreng. Both *In Vitro* and *In Vivo* . Evid. Based Complement. Alternat Med. 2012, 508137. 10.1155/2012/508137 21915187 PMC3170901

[B15] Chuang W. L. Sun Pan B. S. (2011). Anti-stress Effects of Glycine Tomentella Hayata in tilapia: Inhibiting COX-2 Expression and Enhancing EPA Synthesis in Erythrocyte Membrane and Fish Growth. J. Agric. Food Chem. 59, 9532–9541. 10.1021/jf2017308 21732613

[B16] Chung M. C. Peng C. Y. (2008). Compilation of New Family Members of the Asteraceae Family in Taiwan. Taiwan: Endemic Species Research Institute.

[B17] Comrey A. L. (1988). Factor-analytic Methods of Scale Development in Personality and Clinical Psychology. J. Consult Clin. Psychol. 56, 754–761. 10.1037//0022-006x.56.5.754 3057010

[B18] Day S. H. Lin Y. Tsai M. Tsao L. Ko H. Chung M. (2002). Potent Cytotoxic Lignans from Justicia Procumbens and Their Effects on Nitric Oxide and Tumor Necrosis Factor-Alpha Production in Mouse Macrophages. J. Nat. Prod. 65, 379–381. 10.1021/np0101651 11908984

[B19] de Vere N. Rich T. C. G. Trinder S. A. Long C. (2015). DNA Barcoding for Plants. Methods Mol. Biol. 1245, 101–118. 10.1007/978-1-4939-1966-6_8 25373752

[B20] Eddouks M. Ajebli M. Hebi M. (2017). Ethnopharmacological Survey of Medicinal Plants Used in Daraa-Tafilalet Region (Province of Errachidia), Morocco. J. Ethnopharmacol 198, 516–530. 10.1016/j.jep.2016.12.017 28003130

[B21] Editorial Committee (2003). Flora of TaiwanFlora of Taiwan. 2nd ed. Executive Yuan, Taiwan: Council of Agriculture.

[B22] Executive Yuan Committee on Chinese Medicine and Pharmacy (2003). Committee on Chinese Medicine and Pharmacy. Executive Yuan, Taiwan: Department of Health. List of Medicinal Plant Resources in Taiwan.

[B23] Fujian Academy of Medical Sciences (1979). Fujian Materia Medica. Fuji: Fujian University of Traditional Chinese Medicine.

[B24] Gurgel A. P. A. D. da Silva J. G. Grangeiro A. R. S. Oliveira D. C. Lima C. M. P. da Silva A. C. P. (2009). *In Vivo* study of the Anti-inflammatory and Antitumor Activities of Leaves from Plectranthus Amboinicus (Lour.) Spreng (Lamiaceae). J. Ethnopharmacol 125, 361–363. 10.1016/j.jep.2009.07.006 19607901

[B25] Heinrich M. Ankli A. Frei B. Weimann C. Sticher O. (1998). Medicinal Plants in Mexico: Healers’ Consensus and Cultural Importance. Soc. Sci. Med. 47, 1859–1871. 10.1016/s0277-9536(98)00181-6 9877354

[B26] Hsu L. M. (2007). Survey on the Distribution of Ambrosia Artemisiifolia in Taiwan. Taiwan: Taiwan Agricultural Chemicals and Toxic Substances Research Institute.

[B27] Huang S-S. Chen T. Deng J. Pao L. Cheng Y. Chao J. (2020). An Ethnobotanical Study on Qīng-Căo-Chá tea in Taiwan. Front. Pharmacol. 11, 931. 10.3389/fphar.2020.00931 32670061 PMC7329985

[B28] Hung Y. C. (2005). Study on Edible Traditional Chinese Medicinal MaterialsExecutive Yuan Committee on Chinese Medicine and Pharmacy. Executive Yuan, Taiwan: Peilin Publishing HouseDepartment of Health.

[B29] Johnson W. Tchounwou P. B. Yedjou C. G. (2017). Therapeutic Mechanisms of vernonia Amygdalina Delile in the Treatment of Prostate Cancer. Molecules 22, 1594. 10.3390/molecules22101594 PMC5661957 28937624

[B30] Kinmen National Park Headquarters (2005). Records of Battles in Kinmen and Investigation. Taiwan: Kinmen National Park Headquarters.2

[B31] Lee C. Kim S. Eum S. Paik J. Bach T. T. Darshetkar A. M. (2019). Ethnobotanical Study on Medicinal Plants Used by Local Van Kieu Ethnic People of Bac Huong Hoa Nature reserve. Vietnam. J. Ethnopharmacol 231, 283–294. 10.1016/j.jep.2018.11.006 30412749

[B32] Lee T. R. (2010). The Study on Kinmen Residents’ National and Regional Identity. Taiwan: Ming Chuan University.

[B34] Li T. T. (2005). Kinmen History. Taiwan. Taiwan: Cultural Affairs Bureau of Kinmen County.

[B35] Li X. Yang Y. Henry R. J. Rossetto M. Wang Y. Chen S. (2015). Plant DNA Barcoding: from Gene to Genome. Biol. Rev. Camb Philos. Soc. 90, 157–166. 10.1111/brv.12104 24666563

[B36] Li Y. M. Zhu L. Jiang J. G. Yang L. Wang D. Y. (2009). Bioactive Components and Pharmacological Action of Wikstroemia Indica (L.) C.A. Mey and its Clinical Application. Curr. Pharm. Biotechnol. 10, 743–752. 10.2174/138920109789978748 19939213

[B38] Lu C. C. (2005). Survey on Folk Plants in Kinmen. Taiwan. Taiwan: Kinmen National Park.

[B39] Lu C. Y. (2014). Memories and Recognition: Recollections of Training of Women from Kinmen. Taiwan: Showwe Information.

[B40] Lu F. Y. (2011). Flora, Kinmen. Taiwan. Taiwan: Kinmen National Park Headquarters.

[B41] Mesfin K. Tekle G. Tesfay T. (2013). Ethnobotanical Study of Traditional Medicinal Plants Used by Indigenous People of Gemad District, Northern Ethiopia. J. Med. Plants Stud. 1, 32–37.

[B42] Miara M. D. Bendif H. Ait Hammou M. Teixidor-Toneu I. (2018). Ethnobotanical Survey of Medicinal Plants Used by Nomadic Peoples in the Algerian Steppe. J. Ethnopharmacol 219, 248–256. 10.1016/j.jep.2018.03.011 29548971

[B43] Pardo-de-Santayana M. Macía M. J. (2015). The Benefits of Traditional Knowledge. Nature 518, 487–488. 10.1038/518487a 25719661

[B45] Plant science’s human factor (2015). Plant Science’s Human Factor. Nat. Plants 1, 15013. 10.1038/nplants.2015.13 27246770

[B46] Reyes-García V. Guèze M. Luz A. C. Paneque-Gálvez J. Macía M. J. Orta-Martínez M. (2013). Evidence of Traditional Knowledge Loss Among a Contemporary Indigenous Society. Evol. Hum. Behav. 34, 249–257. 10.1016/j.evolhumbehav.2013.03.002 PMC3837211 24277979

[B47] Ribeiro R. V. Bieski I. G. C. Balogun S. O. de Oliveira Martins D. T. (2017). Ethnobotanical Study of Medicinal Plants Used by Ribeirinhos in the North Araguaia Microregion, Mato Grosso, Brazil. J. Ethnopharmacol 205, 69–102. 10.1016/j.jep.2017.04.023 28476677

[B48] Shan S. Huang X. Shah M. H. Abbasi A. M. (2019). Evaluation of Polyphenolics Content and Antioxidant Activity in Edible Wild Fruits. Biomed. Res. Int. 2019, 1381989. 10.1155/2019/1381989 30792989 PMC6354156

[B49] Sung Y. M. (2016). Frontline Island: Kinmen during the Cold War. Taiwan: National Taiwan University Press.

[B50] Sung Y. M. (2009). The Politics of Memory in a Geopolitical Flash Point: Kinmen (Quemoy) 1949–2008. J. Archaeology Anthropol. 71, 41–69.

[B52] Tsai C. C. (2003). Saying Goodbye to Asthma and Allergies *(CS0025)* . Taiwan: China Times Publishing Company.

[B53] Tseng W. H. (2010). Introduction to Kinmen National Park. Taiwan. Taiwan: Kinmen National Park Headquarters.

[B55] Vulić J. J. Čanadanović-Brunet J. M. Ćetković G. S. Djilas S. M. Tumbas Šaponjac V. T. Stajčić S. S. (2016). Bioactive Compounds and Antioxidant Properties of Goji Fruits (Lycium Barbarum L.) Cultivated in Serbia. J. Am. Coll. Nutr. 35, 692–698. 10.1080/07315724.2016.1142404 27710210

[B56] Wang B. S. Juang L. Yang J. Chen L. Tai H. Huang M. (2012). Antioxidant and Antityrosinase Activity of Flemingia Macrophylla and Glycine Tomentella Roots. Evid. Based Complement. Alternat Med. 2012, 431081. 10.1155/2012/431081 22997529 PMC3444970

[B58] Wang Y. W. Chuang J. Chang T. Won S. Tsai H. Lee C. (2015). Antiangiogenesis as the Novel Mechanism for Justicidin A in the Anticancer Effect on Human Bladder Cancer. Anti Cancer Drugs 26, 428–436. 10.1097/CAD.0000000000000203 25569706

[B59] Wu D. Zhang X. Liu L. Guo Y. (2019). Key CMM Combinations in Prescriptions for Treating Mastitis and Working Mechanism Analysis Based on Network Pharmacology. Evid. Based Complement. Alternat Med. 2019, 8245071. 10.1155/2019/8245071 30911319 PMC6399531

[B60] Wu H. T. (2007). Plant Use Knowledge of Hakka Ethnobotany in Dahu Township. Miaoli County. Taiwan: National Pingtung University of Science and Technology.

[B61] Wu K. C. Lin W. Sung Y. Wu W. Cheng Y. Chen T. (2019). Glycine Tomentella Hayata Extract and its Ingredient Daidzin Ameliorate Cyclophosphamide-Induced Hemorrhagic Cystitis and Oxidative Stress through the Action of Antioxidation, Anti-fibrosis, and Anti-inflammation. Chin. J. Physiol. 62, 188–195. 10.4103/CJP.CJP_60_19 31670282

[B62] Wu L. C. (2014). Comparison of Convenience Sampling and Purposive Sampling. J. Nurs. 61, 3–105. 10.6224/JN.61.3.105 24899564

[B63] Wu S. T. (2004). Ethnobotany of Kinmen. Taiwan: National Chung Hsing University.

[B64] Wu Z. Yang L. He L. Wang L. Peng L. (2020). Systematic Elucidation of the Potential Mechanisms of Core Chinese Materia Medicas in Treating Liver Cancer Based on Network Pharmacology. Evid. Based Complement. Alternat Med. 2020, 4763675. 10.1155/2020/4763675 32382293 PMC7196158

[B65] Xu X. Y. Wang D. Ku C. Zhao Y. Cheng H. Liu K. (2019). Anti-HIV Lignans from Justicia Procumbens. Chin. J. Nat. Med. 17, 945–952. 10.1016/S1875-5364(19)30117-7 31882050

[B66] Yemele M. D. Telefo P. B. Lienou L. L. Tagne S. R. Fodouop C. S. P. Goka C. S. (2015). Ethnobotanical Survey of Medicinal Plants Used for Pregnant Womens Health Conditions in Menoua Division-West Cameroon. J. Ethnopharmacol 160, 14–31. 10.1016/j.jep.2014.11.017 25449451

[B67] Yen H. F. (2010). Summary of Traditional Crude Drugs of the Amis People. Taiwan. Taiwan: National Museum of Natural Science.

[B68] Yen J. H. Yang D. Chen M. Hsieh Y. Sun Y. Tsay G. J. (2010). Glycine Tomentella Hayata Inhibits IL-1beta and IL-6 Production, Inhibits MMP-9 Activity, and Enhances RAW264.7 Macrophage Clearance of Apoptotic Cells. J. Biomed. Sci. 17, 83. 10.1186/1423-0127-17-83 21054849 PMC2988746

[B69] Zhang Y. Bao F. Hu J. Liang S. Zhang Y. Du G. (2007). Antibacterial Lignans and Triterpenoids from Rostellularia Procumbens. Planta Med. 73, 1596–1599. 10.1055/s-2007-993747 18058608

