# Corrigendum: An Ethnobotanical Study of Medicinal Plants in Kinmen

**DOI:** 10.3389/fphar.2022.873011

**Published:** 2022-03-09

**Authors:** Shyh-Shyun Huang, Chia-Hung Huang, Chien-Yu Ko, Ting-Yang Chen, Yung-Chi Cheng, Jung Chao

**Affiliations:** ^1^ School of Pharmacy, China Medical University, Taichung, Taiwan; ^2^ Department of Food Nutrition and Health Biotechnology, Asia University, Ministry of Health and Welfare, Taichung, Taiwan; ^3^ Department of Pharmacy, Kinmen Hospital, Ministry of Health and Welfare, Kinmen, Taiwan; ^4^ Department of Nursing, National Quemoy University, Kinmen, Taiwan; ^5^ Department of Pharmacology, Yale University School of Medicine, New Haven, CT, United States; ^6^ Chinese Medicine Research Center, Department of Chinese Pharmaceutical Sciences and Chinese Medicine Resources, Master Program for Food and Drug Safety, China Medical University, Taichung, Taiwan

**Keywords:** ethnobotany, historical source, field investigation, quemoy traditional medicine, Taiwan traditional medicine

In the original article, there was a mistake in Table 1 as published. The corrected Table 1 appears below.

**TABLE 1:** The important historical events that promoted ethnobotanical plant exchange in Kinmen.

The Eastern Jin Dynasty (A.D. 317–420)	Since the occurrence of frequent wars in the central plains during the Eastern Jin Dynasty (A.D. 317–420), people have taken refuge and moved to Kinmen because of its special geographical location
	Therefore, the migrant population has influenced the use of plants in the kinmen area for the first time in history (Li, 2009)
The Ming Zheng period (A.D. 1662–1683)	In the Ming Zheng period (A.D. 1662–1683), Zheng Chenggong took Kinmen as the base for rebelling against the Qing Dynasty and rebuilding the Ming Dynasty
	Therefore, a large number of people in Southern Fujian migrated to Kinmen along with the army (Li, 2005)
The Qing Dynasty (A.D. 1820–1850)	After the Opium War in the Daoguang Period of the Qing Dynasty (A.D. 1820–1850), five ports in China were opened for international trading and a large number of native people moved to Southeast Asia, making Kinmen the hometown of Southern Fujian culture as well as the hometown of overseas Chinese This spread the use of Kinmen plants overseas and introduced Southern Fujian culture and Nanyang folk customs to Kinmen (Li, 2009)
The period of Republic of China (A.D. 1911–1945)	After the Revolution of 1911 (A.D. 1911), China was governed by the Republic of China. At that time, Kinmen belonged to the Republic of China, while Taiwan was under the jurisdiction of Japan (Lee, 2010)
	During this period, the Japanese conducted a complete ethnobotanical survey of Taiwan, including plants used by the indigenous people
The civil war between the Kuomintang and the Communist Party (A.D. 1945–1950)	Before the end of the civil war between the Kuomintang and the Communist Party (A.D. 1950), life in Kinmen was closely connected with that in Fujian Province, but after the end of this civil war, the Republic of China withdrew to Taiwan, and Kinmen became the front line for defending the country During the Civil War between Kuomintang and the Communist Party, the influence of the war led to Kinmen establishing close and sometimes antagonistic relations with China and Taiwan, but the exchanges between soldiers from other provinces and Kinmen residents were more frequent This state brought more traditional medical knowledge into Kinmen
The period of Republic of China in Taiwan (since A.D. 1949)	The Millennium (A.D. 2000) marked the opening of Mini Three Links (Fujian Province of the People’s Republic of China and Kinmen County of Fujian Province of the Republic of China became engaged in trade, postal service, and navigation; mainland people were allowed to travel between China and Taiwan via Kinmen). It not only affected food, clothing, housing, and transportation, but also increased the cultural exchanges of Kinmen ethnic groups, thus affecting the residents’ ways of using plants (Wu, 2004)

Furthermore, a word was misused in the text of the introduction. A correction has been made to **Introduction**, Paragraph 1:

“Kinmen (Quemoy) is located on the west coast of the Pacific Ocean, specifically in the west-central island area of Taiwan’s main island (Chen, 2017). At present, its correct legal name is Kinmen County, and it is under the jurisdiction of Fujian Province, the Republic of China. A narrow part of the sea separates it from Xiamen City in Fujian Province, making it an independent island surrounded by the sea.”

There was also an error in affiliation 6. Instead of “Chinese Medicine Research Center, Department of Chinese Pharmaceutical Sciences and Chinese Medicine Resources, China Medical University, Taichung, Taiwan”, it should be “Chinese Medicine Research Center, Department of Chinese Pharmaceutical Sciences and Chinese Medicine Resources, Master Program for Food and Drug Safety, China Medical University, Taichung, Taiwan”.

The authors apologize for this error and state that this does not change the scientific conclusions of the article in any way. The original article has been updated.

