# Supplementary material for: An Ethnobotanical Study of Medicinal Plants in Kinmen
Source: Front Pharmacol. 2022 Feb 9;12:681190. doi: 10.3389/fphar.2021.681190 (PMC8864234; doi:10.3389/fphar.2021.681190)
Supplement: Supplementary file 1 [file DataSheet1.docx]

**Table S1 Research team's survey date and number of informants in Kinmen**

| **Date** | **Township of Investigation** | **Male (Accept interview)** | **Woman (Accept interview)** | **No experience in using medicinal plants (No interviews)** |
| --- | --- | --- | --- | --- |
| 08.31.2019 | Kinhu Town | 3 | 2 | 1 |
| 09.07.2019 | Kinhu Town | 4 | 5 | 0 |
| 09.15.2019 | Kinsha Town | 2 | 5 | 0 |
| 09.28.2019 | Kinsha Town | 6 | 6 | 0 |
| 10.06.2019 | Jinning Township | 5 | 4 | 1 |
| 10.12.2019 | Jinning Township | 4 | 2 | 0 |
| 10.22.2019 | Jincheng Town | 3 | 2 | 3 |
| 11.07.2019 | Jincheng Town | 2 | 5 | 0 |
| 11.23.2019 | Kinhu Town | 3 | 5 | 0 |
| 11.30.2019 | Lieyu Township | 2 | 4 | 2 |
| 12.05.2019 | Jincheng Town | 1 | 3 | 1 |
| 12.17.2019 | Lieyu Township | 0 | 2 | 0 |

Table S2 Basic data of medicinal plants in the results of this study

| No | Family | Scientific name  Voucher specimen number | | Local name | Pars used | Preparation method | ΣUi | UV | FC | RFC | Ailments |
| --- | --- | --- | --- | --- | --- | --- | --- | --- | --- | --- | --- |
| 1 | Acanthaceae | *Andrographis paniculata* (Burm. f.) Nees  CMUK047 | | Kǔ Chá | Le | Decoction  /Oral | 6 | 0.08 | 5 | 0.06 | Detoxification |
| 2 |  | *Dicliptera chinensis* (L.) Juss.  CMUK018 | | Lioù Jiaǒ Ying | Wp | Decoction  /Oral | 4 | 0.05 | 4 | 0.05 | Clear heat and resolve toxin, hepatitis |
| 3 |  | *Justicia procumbens* L.  CMUK008 | | Yi Ạ Tsaǒ | Wp | Decoction  /Oral | 74 | 0.93 | 72 | 0.9 | Allergic rhinitis, cold and cough, sore throat |
| 4 |  | *Rhinacanthus nasutus* (L.) Kurz  CMUK006 | | Baí Hè Tsaǒ | Wp | Decoction  /Oral | 4 | 0.05 | 2 | 0.03 | Hepatitis |
| 5 | Amaranthaceae | *Achyranthes aspera* var. rubrofusca (Wight) Hook.f.CMUK013 | | Tiěh Mǎ Bian | St&Le | Fry/Oral | 2 | 0.03 | 2 | 0.03 | Joint pain, activating blood |
| 6 | Amaryllidaceae | *Crinum asiaticum* L.  CMUK028 | | Yǐn Shueǐ Jiao | Le | Raw/ Fomentation | 8 | 0.1 | 8 | 0.1 | Skin redness |
| 7 |  | *Allium fistulosum* L.  CMUK050 | | Huang Tsang | St | Cook with meat/Oral | 4 | 0.05 | 2 | 0.03 | Cough, chillness |
| 8 |  | *Allium tuberosum* Rottler ex Spreng.  CMUK048 | | Jioǔ Tsaì Zǐh | Se | Burn/  Filling teeth | 1 | 0.01 | 1 | 0.01 | General and unspecified |
| 9 | Annonaceae | *Annona squamosa* L.  CMUK015 | | Shìh Jia | Le | Soak/Bath | 1 | 0.01 | 1 | 0.01 | Skin rash, reddish |
| 10 | Apocynaceae | *Strophanthus divaricatus* (Lour.) Hook. & Arn.  CMUK022 | | Nioú Jiaǒ Téng | Le | Crush/Apply to injuries | 4 | 0.05 | 4 | 0.05 | Bruises |
| 11 | Aristolochiaceae | *Aristolochia kaempferi* Willd.  CMUK065 | | Lí La Tsaǒ | Ro | Crush/Apply to injuries | 4 | 0.05 | 3 | 0.04 | Herpes |
| 12 | Aspleniaceae | *Asplenium nidus* L.  CMUK046 | | Chaó Jyuéh | Wp | Decoction/Oral | 2 | 0.03 | 2 | 0.03 | Bruises |
| 13 | Basellaceae | *Anredera cordifolia* (Ten.) Steenis  CMUK002 | | Chuan Chi/  Yún Nán Baí Yaò | Le | Crush/Apply to injuries | 8 | 0.1 | 8 | 0.1 | Hemorrhage |
| 14 | Boraginaceae | *Heliotropium strigosum* Willd. CMUK054 | |  | Wp | Decoction/Oral | 3 | 0.04 | 2 | 0.03 | Clear heat and resolve toxin |
| 15 | Brassicaceae | *Raphanus raphanistrum* L.  CMUK012 | | Tsaì Toú Yèh | Le | Decoction/  Fomentation | 6 | 0.08 | 4 | 0.05 | Frostbite |
| 16 | Cactaceae | *Epiphyllum oxypetalum* (DC.) Haw.  CMUK080 | | Chyóng Hua | Fl | Honey maceration/  Oral | 4 | 0.05 | 4 | 0.05 | Sore throat |
| 17 | Caryophyllaceae | *Silene aprica* Turcz.  CMUK076 | | Lí Loǔ Hua | Wp | Decoction/Oral | 2 | 0.03 | 1 | 0.01 | Activating blood |
| 18 | Commelinaceae | *Tradescantia pallida (Rose)* D. R. Hunt  CMUK041 | | Rú Yì Lán | Wp | Decoction/Oral | 4 | 0.05 | 2 | 0.03 | Urinary tract infection |
| 19 |  | *Tradescantia spathacea* Sw*.*  CMUK001 | | Hóng Jhú Yèh | Le | Decoction/Oral | 33 | 0.41 | 20 | 0.25 | Allergic rhinitis, cold, clear heat and resolve toxin |
| 20 | Compositae | *Artemisia argyi* H.Lév.& Vaniot  CMUK021 | |  | Le | Crush/Apply to injuries | 5 | 0.06 | 4 | 0.05 | Hemorrhage |
| 21 |  | *Bidens bipinnata* L.  CMUK004 | | Shén Jhen Tsaǒ | Wp | Decoction/Oral | 10 | 0.13 | 6 | 0.08 | Clear heat and resolve toxin, gout |
| 22 |  | *Bidens pilosa* L.  CMUK077 | | Siaò Chá Moǔ | Wp | Decoction/Oral | 12 | 0.15 | 7 | 0.09 | Clear heat and resolve toxin, diabetes, diarrhea |
| 23 |  | *Chrysanthemum morifolium* Ramat.  CMUK059 | | Jyú Hua | Le | Decoction/Oral | 8 | 0.1 | 6 | 0.08 | Eye disease, dizziness |
| 24 |  | *Cirsium japonicum* (Thunb.) Fisch. ex DC.  CMUK036 | | Ji Jiaǒ Jì | Wp | Decoction/Oral | 12 | 0.15 | 7 | 0.09 | Liver diseases, diabetes |
| 25 |  | *Crossostephium chinense* (A. Gray ex L.) Makino  CMUK005 | | Haǐ Fú Róng | Wp | Decoction/Oral | 8 | 0.1 | 6 | 0.08 | Rheumatism, joint pain |
| 26 |  | *Tithonia diversifolia* (Hemsl.) A. Gray  CMUK024 | | Wáng Yéh Kueí | St | Decoction/Oral | 4 | 0.05 | 2 | 0.03 | Clear heat and resolve toxin, liver diseases |
| 27 |  | *Tridax procumbens* (L.)L.  CMUK043 | | Feì Yán Tsaǒ | Wp | Decoction/Oral | 2 | 0.03 | 2 | 0.03 | Cough |
| 28 |  | *Vernonia amygdalina* Delile  CMUK049 | | Gan Lián | Le | Soak/Oral | 7 | 0.09 | 7 | 0.09 | Hepatitis |
| 29 |  | *Wedelia prostrata* Hemsl.  CMUK009 | | Shé Tsaǒ | Wp | Decoction/Oral | 3 | 0.04 | 2 | 0.03 | Bruise, swollen |
| 30 | Convolvulaceae | *Cuscuta campestris* Yunck.  CMUK074 | | Wú Gen Tsaǒ | Wp | Decoction/Oral | 2 | 0.02 | 14 | 0.18 | Promoting urination, kidney disease |
| 31 | Crassulaceae | *Bryophyllum delagoense* (Eckl. & Zeyh.) Druce  CMUK033 | | Dan Tsaǒ | Wp | Decoction/Oral | 1 | 0.01 | 1 | 0.01 | Hemorrhagic dengue fever |
| 32 |  | *Graptopetalum paraguayense* (N.E.Br.) E. Walther  CMUK072 | | Shíh Lián Hua | Le | Raw/Oral &  Juice/Apply to skin | 26 | 0.33 | 20 | 0.25 | Liver diseases, sore throat  antipruritic |
| 33 |  | *Orostachys fimbriata* (Turcz.) A. Berger CMUK070 | | Jian Dan Tsaǒ | Le | Crush/Apply to injuries | 1 | 0.01 | 1 | 0.01 | Wound healing, reddish, abruption |
| 34 | Cucurbitaceae | *Momordica charantia* L.  CMUK067 | | Shan Kǔ Gua | Fr | Decoction/Oral | 12 | 0.15 | 8 | 0.1 | Clear heat and resolve toxin, diabetes |
| 35 | Dioscoreaceae | *Dioscorea alata* L.  CMUK039 | | Shan Yaò | St | Decoction/Oral | 8 | 0.1 | 6 | 0.08 | Digestion, stomach disorder |
| 36 | Euphorbiaceae | *Euphorbia hirta* L. CMUK025 | | Jhu Mǔ Tsaì | Wp | Decoction/Apply to skin | 2 | 0.03 | 2 | 0.03 | Athlete’s foot |
| 37 |  | *Euphorbia thymifolia* L.  CMUK073 | | Rǔ Zaǐ Tsaǒ | Wp | Decoction/Oral | 5 | 0.06 | 4 | 0.05 | Flatulence, constipation |
| 38 |  | *Jatropha curcas* L.  CMUK027 | | Baí Tǔ Pí | St&Le | Juice/Apply to injuries | 16 | 0.2 | 13 | 0.16 | Recurrent aphthous stomatitis, herpes |
| 39 | Krameriaceae | *Krameria prostrata* Brandegee  CMUK052 | | Tsìh Chioú | Fr | Decoction/Oral | 3 | 0.04 | 2 | 0.03 | Sore throat |
| 40 | Lamiaceae | *Leucas chinensis* (Retz.) Sm.  CMUK030 | | Baí Hua Tsaǒ | Wp | Decoction/Oral | 2 | 0.03 | 1 | 0.01 | Diarrhea |
| 41 |  | *Mentha spicata* L. CMUK020 | | Bò Hé | Le | Soak or Decoction/Oral | 12 | 0.15 | 9 | 0.11 | Headache, Sore throat |
| 42 |  | *Ocimum basilicum* L.  CMUK003 | | Jioǔ Tséng Tǎ | Le & Ro | Le: Fry/Chew  Ro: Cook with meat/Oral | 8 | 0.1 | 6 | 0.08 | Blood purifier |
| 43 |  | *Plectranthus amboinicus* (Lour.) Spreng.  CMUK007 | | Zuǒ Shoǔ Siang | Le | Crush/Apply to injuries | 22 | 0.28 | 20 | 0.25 | Contusion, joint pain, reddish |
| 44 |  | *Scutellaria barbata* D. Don  CMUK062 | | Bàn Jhih Lián | Wp | Decoction/Oral | 3 | 0.04 | 2 | 0.03 | Hepatitis |
| 45 | Leguminosae | *Glycine tomentella* Hayata.  CMUK010 | | Yì Tiaó Gen | Ro | Soak or Decoction/Oral | 36 | 0.45 | 33 | 0.41 | Rheumatism, joint pain, contusion, reddish |
| 46 |  | *Mimosa pudica* L. CMUK053 | | Jiàn Siaò Tsaǒ | Wp | Decoction/Oral | 2 | 0.03 | 1 | 0.01 | Allergic rhinitis, cold and cough, running nose, sputum |
| 47 |  | *Senna occidentalis (L.)* Link  CMUK055 | | Shan Kafei | Se | Decoction/Oral | 8 | 0.1 | 6 | 0.08 | Eye diseases |
| 48 |  | *Senna tora* (L.) Roxb.  CMUK017 | | Jyuéh Míng | Se | Decoction/Oral | 12 | 0.15 | 9 | 0.11 | Promoting urination, eye diseases |
| 49 | Lindsaeaceae | *Odontosoria chinensis* (L.) J. Sm. CMUK051 | | Ní | Wp | Decoction/Oral | 1 | 0.01 | 1 | 0.01 | Promoting urination |
| 50 | Lygodiaceae | *Lygodium japonicum* (Thunb.) Sw.  CMUK057 | | Jhen Jhong Maó | Sp | Crush/Apply to skin | 2 | 0.03 | 2 | 0.03 | Shingles |
| 51 | Lythraceae | *Lawsonia inermis* L.  CMUK082 | | Jhǐh Jiǎ Hua | Le | Crush/Apply to injuries | 2 | 0.03 | 2 | 0.03 | Sprain, swollen |
| 52 | Malvaceae | *Hibiscus sabdariffa* L.  CMUK045 | | Luò Shén Hua | Fl(Calyx) | Soak or Decoction/Oral | 2 | 0.03 | 2 | 0.03 | Edema |
| 53 | Meliaceae | *Toona sinensis* (Juss.) M. Roem.  CMUK058 | | Siangjhuang | Le | Decoction/Oral | 1 | 0.01 | 1 | 0.01 | Clear heat and resolve toxin, promoting urination |
| 54 | Moraceae | *Morus alba* L.  CMUK044 | | Suan Chaí | Fr | Raw or Decoction/Oral | 27 | 0.34 | 20 | 0.25 | Anemia |
| 55 | Myoporaceae | *Pentacoelium bontioides* Siebold & Zucc.  CMUK011 | | Kǔ Lán Pán | Wp | Crush/Apply to injuries | 1 | 0.01 | 1 | 0.01 | Reddish, swollen |
| 56 | Myrtaceae | *Psidium guajava* L. CMUK060 | | Ná A Pú | Le | Soak /Oral | 2 | 0.03 | 2 | 0.03 | Diarrhea, diabetes |
| 57 | Oleaceae | *Osmanthus fragrans* Lour.  CMUK056 | | Gueì Hua | Fl | Soak or Decoction/Oral | 6 | 0.08 | 4 | 0.05 | Sputum |
| 58 | Orchidaceae | *Cymbidium ensifolium* (L.) Sw. CMUK037 | | Jiàn Lán | Le | Decoction/Oral | 2 | 0.03 | 1 | 0.01 | Cough |
| 59 | Oxalidaceae | *Oxalis debilis* var. corymbosa (DC.) Lourteig  CMUK031 | | Roù Zòng Tsaǒ | Wp&  St(Bulb) | St: Decoction, Wp: Crush/Oral | 3 | 0.04 | 2 | 0.03 | Fever, sore throat, promoting urination |
| 60 | Plantaginaceae | *Plantago asiatica* L.  CMUK075 | | Wǔ Jin Tsaǒ | Wp | Decoction/Oral | 21 | 0.26 | 16 | 0.2 | Flatulence, cough, bronchitis |
| 61 | Poaceae | *Eleusine indica* (L.) Gaertn*.*  CMUK081 | | Nioú Dùn Tsaǒ | Wp | Decoction/Oral | 2 | 0.03 | 1 | 0.01 | Nausea, vomiting, fatigue, diarrhea |
| 62 |  | *Imperata cylindrica* (L.) Raeusch  CMUK068 | | Mǎ Tsaǒ Gen | Wp | Decoction/Oral | 24 | 0.3 | 20 | 0.25 | Fever, cold, myalgia |
| 63 | Portulaceace | *Portulaca oleracea* L.  CMUK069 | | Jhu Mǔ Rǔ | Le | Decoction/Oral | 3 | 0.04 | 2 | 0.03 | Cardiovascular |
| 64 | Pteridaceae | *Pteris ruticose* Poir CMUK019 | | Ji Jiaǒ Tsaǒ | Wp | Decoction/Oral | 2 | 0.03 | 2 | 0.03 | Clear heat and resolve toxin, diarrhea |
| 65 | Rosaceae | *Agrimonia pilosa* Ledeb.  CMUK016 | | Sian Hè Tsaǒ | Wp | Crush/Oral | 1 | 0.01 | 1 | 0.01 | Respiratory system disease |
| 66 |  | *Eriobotrya japonica* (Thunb.) Lindl.  CMUK035 | | Pí Pá | Le | Decoction/Oral | 2 | 0.03 | 2 | 0.03 | Sputum, cough |
| 67 |  | *Potentilla discolor* Bunge  CMUK014 | | Guó Sìng Tsaǒ | Wp | Soak /Oral | 1 | 0.01 | 1 | 0.01 | Fever |
| 68 |  | *Rubus parvifolius* L.  CMUK063 | | Hǔ Meí Tsìh | Fr | Raw/Oral; Juice/Apply to skin | 4 | 0.05 | 2 | 0.03 | Cloudy urine, antipruritic |
| 69 | Rubiaceae | *Oldenlandia diffusa* (Willd.) Roxb.  CMUK040 | | Jhu Zaǐ Tsaǒ | Wp | Decoction/Oral | 3 | 0.04 | 2 | 0.03 | Clear heat and resolve toxin, cancer |
| 70 | Rutaceae | *Citrus japonica* Thunb. CMUK026 | | Jin Jiéh | Peel | Decoction/Oral | 2 | 0.03 | 2 | 0.03 | Cold and cough, sore throat |
| 71 | Sapindaceae | *Euphoria longana* Lam  CMUK064 | | Gueì Yuán | Fr | Decoction/Oral | 4 | 0.05 | 4 | 0.05 | Insomnia, fatigue |
| 72 | Saururaceae | *Houttuynia cordata* Thunb*.*  CMUK042 | | Jí | Le | Decoction/Oral | 2 | 0.03 | 2 | 0.03 | Cough, sputum |
| 73 | Saxifragaceae | *Saxifraga stolonifera* Curtis  CMUK032 | | Hoú Ní Tsaǒ | Le | Crush/Apply to injuries | 1 | 0.01 | 1 | 0.01 | Reddish, swollen |
| 74 | Solanaceae | *Datura stramonium* L.  CMUK078 | | Màn Tuó Luó | Se | Burn/ Fumigation | 1 | 0.01 | 1 | 0.01 | Hemorrhoids |
| 75 |  | *Lycium chinense* Mill. CMUK066 | | Koǔ Ní Tsan  Gan Goǔ Chǐ | Ro & Fr | Decoction/Oral | 32 | 0.4 | 18 | 0.23 | Fever, heat stroke, eye disease |
| 76 |  | *Physalis ruticos* L.  CMUK071 | | Paò Zaǐ Tsaǒ | Wp | Decoction/Oral | 4 | 0.05 | 4 | 0.05 | Allergic rhinitis, cough |
| 77 |  | *Solanum incanum* L.  CMUK083 | | Huáng Shueǐ Yaó | St | Decoction/Oral | 2 | 0.03 | 2 | 0.03 | Hepatitis |
| 78 |  | *Solanum violaceum* Ortega  CMUK023 | | Nioǔ Zaǐ Jia | Ro | Decoction/Oral | 1 | 0.01 | 1 | 0.01 | Hepatitis |
| 79 | Thymelaeaceae | *Wikstroemia indica* (L.) C.A. Mey. CMUK034 | | Pǔ Lún | Wp | Crush/Apply to injuries | 6 | 0.08 | 5 | 0.06 | Athlete’s foot, reddish, bruise |
| 80 | Verbenaceae | *Phyla nodiflora* (L.) Greene  CMUK038 | | Ya Shé Hóng | Le | Crush/Oral | 18 | 0.22 | 13 | 0.16 | Sore throat |
| 81 |  | *Verbena officinalis* L.  CMUK029 | | Mǎ Bian Tsaǒ | Wp | Decoction/Oral | 1 | 0.01 | 1 | 0.01 | Diuretic |
| 82 | Xanthorrhoeaceae | | *Aloe vera* (L.) Burm.f.  CMUK061 | Lú Hueì | Le | Juice/Apply to skin | 3 | 0.04 | 3 | 0.04 | Reddish, antipruritic |
| 83 | Zygophyllaceae | *Tribulus terrestris L.*  CMUK079 | | San Jiaǒ Mǎ Zaǐ | Fr | Raw/Oral | 4 | 0.05 | 3 | 0.04 | Headache, dizziness |
